# Development of thin-film micro-outlets for spatially constraining local PO_2_ perturbations to capillaries *in vivo*

**DOI:** 10.3389/fphys.2025.1575776

**Published:** 2025-07-09

**Authors:** Meghan E. Kiley, Richard J. Sové, Reilly H. Smith, Brenda N. Wells, Gaylene M. Russell McEvoy, Graham M. Fraser

**Affiliations:** ^1^ Division of BioMedical Sciences, Faculty of Medicine, Memorial University of Newfoundland, St. John’s, NL, Canada; ^2^ Department of Biomedical Engineering, School of Medicine, Johns Hopkins University, Baltimore, MD, United States

**Keywords:** microcirculation, oxygen transport, blood flow regulation, capillary hemodynamics, microfluidic device

## Abstract

**Objective:**

To develop and validate thin-film micro-outlet devices to study microvascular blood flow responses to localized changes in skeletal muscle oxygen concentration ([O_2_]).

**Methods:**

30 male Sprague-Dawley rats (159–194 g) were anesthetized and instrumented to maintain cardiovascular state. The extensor digitorum longus (EDL) muscle was dissected, isolated, and reflected over a gas exchange chamber (GEC) mounted in the stage of an inverted microscope. The GEC and EDL were coupled via a composite, gas permeable membrane, and a gas impermeable film fabricated with laser machined micro-outlets of specific diameters (200, 400, 600, and 1,000 μm). [O_2_] in the EDL was dynamically manipulated with step-wise oscillations between 7% (1 min) → 12% (1 min) → 2% (1 min) → 7% (1 min), and step challenges from 7% (1 min) → 2% or 12% (2 min), while recording intravital video for capillary RBC oxygen saturation (SO_2_) and hemodynamic measurements. Oxygen diffusion between tissue and micro-outlet devices was modelled using a finite element mass transport model to further validate experimental results.

**Results:**

[O_2_] oscillations imposed on capillaries directly overlying 400 μm micro-outlets caused significant changes in RBC SO_2_ at 12% and 2% [O_2_], compared to 7% [O_2_] (p < 0.0001). [O_2_] oscillations caused significant changes in capillary RBC supply rate (SR) at 2% [O_2_] versus 7%, and were significantly different at 2% compared to 12% [O_2_] (p < 0.0014). Similarly, [O_2_] challenges imposed on capillaries overlying 200 μm micro-outlets also caused significant changes in RBC SO_2_ at 2% [O_2_], compared to 7% [O_2_] (p < 0.0001), and caused significant changes in SR at 2% [O_2_] compared to 7% (p < 0.0001).

**Conclusion:**

Our composite thin-film devices were fabricated and validated to spatially confine oxygen perturbations to capillaries using micro-outlets of varying diameters. These results demonstrate that our devices can manipulate capillary SO_2_ and alter capillary RBC SR in vessels directly overlying the micro-outlet without affecting capillary SO_2_ at a distance from the outlets. Our novel composite thin-film micro-outlet devices demonstrate that capillary blood flow responses can be provoked by manipulating [O_2_] in tissue regions as small as ∼200 μm in diameter.

## 1 Introduction

Oxygen (O_2_) mediated blood flow regulation is essential for meeting the metabolic demands associated with body organs and tissues (Duling and Berne, 1970a; Duling and Klitzman, 1980; Segal, 2005; Golub and Pittman, 2013). Under normal physiological conditions, energy is produced by oxidative phosphorylation in skeletal muscle to synthesize ATP, a process that requires a constant oxygen supply. To match ever-changing local oxygen demands in tissues, the arteriolar wall will vasodilate in response to low tissue O_2_ concentration ([O_2_]) to increase blood flow into capillary beds or vasoconstrict in response to high tissue [O_2_] (Duling, 1972; Pittman and Duling, 1973; Duling, 1974; Duling, 1973; Hutchins et al., 1974; Sullivan and Johnson, 1981; Jackson, 1986; Jackson et al., 1987; Dietrich et al., 2000; Ellis et al., 2005; Ngo et al., 2010).

The regulation of oxygen-mediated blood flow is highly localized and primarily driven by conditions within tissue microenvironments (Sparks, 1980; Renkin, 1985; Golub and Pittman, 2013). Regulatory mechanisms responsible for sensing flow changes to wall shear stress or vascular resistance from wall tension do not directly sense O_2_ concentration levels or precisely regulate O_2_ supply (Ellis et al., 2012; Schubert and Mulvany, 1999; Pohl et al., 2000; Carlson and Secomb, 2005). Several local mechanisms are likely responsible for this active regulation, and several have been proposed (as reviewed in Jackson, 2016). However, existing evidence fails to account for the sensitivity of vascular responses across the physiological range of tissue [O_2_] and at different scales (Duling and Berne, 1970b; Jackson and Duling, 1983; Messina et al., 1994; Pries et al., 1986; Ellsworth et al., 1995; Ellsworth et al., 2009; Jackson, 2016). Studies have been conducted over a wide range of partial pressure of oxygen (PO_2_) conditions (10–150 mmHg) where some may not have physiological relevance at the site they are observed (Coburn et al., 1979; Tateishi and Faber, 1995a; Tateishi and Faber, 1995b; Kerkhof et al., 1999; Jackson, 2016).

Oxygen reactivity studies have been completed on various vessels along the vascular tree using a range of experimental techniques. These varied approaches may impact the responses observed depending on the vessel of interest, as described below. Evidence exists for potential O_2_ sensors, including the parenchymal tissue, components of the vascular wall, and the red blood cells (Duling and Berne, 1970a; Pittman and Duling, 1973; Jackson and Duling, 1983; Hester, 1993; Pries et al., 1986; Ellsworth et al., 1995; Ellsworth, 2004; Sprague et al., 2009; Jackson, 2016). Controversial evidence exists on their involvement in arteriolar O_2_ regulation, but in the absence of the parenchyma in arterioles, O_2_ responses are blunted (Jackson and Duling, 1983; Harder et al., 1996). There are *ex vivo* results that support vascular smooth muscle cells as the O_2_ sensor, but when the endothelium is removed from arterioles and feed arteries, O_2_ reactivity becomes blunted or diminished entirely (Frisbee et al., 2002; Messina et al., 1992; Tateishi and Faber, 1995a; Tateishi and Faber, 1995b; Kerkhof et al., 1999). Additionally, *in vivo* studies on hamster cheek pouches provided evidence that none of the arteriolar wall components directly sense O_2_ changes (Jackson et al., 1987; Duling, 1974). However, data collected by pressure myography supports endothelial cells’ participation in the O_2_ reactivity response (Busse et al., 1983; 1984; Pohl and Busse, 1989; Jackson et al., 1987; Messina et al., 1992; 1994; Fredricks et al., 1994; Ward, 1999; Frisbee, 2001; Frisbee and Stepp, 2001; Frisbee et al., 2001; Frisbee et al., 2002). Considering the differences in the vasculature used to obtain this experimental evidence is essential. *Ex vivo* studies, for example, have focused on first-order arterioles and arteries, whereas intravital studies utilize small, third-to-fifth-order arterioles. Given the range of contemporary methodologies, it is important to develop appropriate experimental techniques to study microvascular blood flow regulation to confirm oxygen-associated mechanisms without interacting with responses in higher-order vessels.

Strong evidence exists in the literature to support red blood cells (RBCs) as the O_2_ sensor (Bergfeld and Forrester, 1992; Stein and Ellsworth, 1993; Ellsworth et al., 1995; Ellsworth, 2004; Ellsworth et al., 2009; Jia et al., 1996; Stamler et al., 1997; Patel et al., 1999; Jagger et al., 2001). RBCs have the cellular machinery to detect PO_2_ changes and initiate regulatory mechanisms, as they are mobile carriers of O_2,_ and have been shown to release ATP, a vasodilatory molecule capable of increasing blood flow in response to O_2_ depletion (Delp, 1999; Olsson, 1981; Bergfeld and Forrester, 1992; Rowell, 2004; Ellsworth et al., 1995; Jagger et al., 2001; Laughlin et al., 2012). It has been proposed that RBCs regulatory actions are initiated by hemoglobin conformational changes that occur when there is a reduction in RBC SO_2_ (Bergfeld and Forrester, 1992; Stein et al., 1993; Ellsworth et al., 1995; Jagger et al., 2001; Buehler and Alayash, 2004; Ellsworth et al., 2009). This allosteric alteration initiates a signal transduction pathway in RBCs, increasing ATP release and transportation through pannexin-1 channels to bind P_2_Y_2_ receptors on the vascular endothelium (Sprague et al., 1996; McCullough et al., 1997; Collins et al., 1998; Dietrich et al., 2000). Then, an upstream vasodilatory response occurs by triggering hyperpolarization conducted via gap junctions between endothelial cells, transducing an ATP signal from capillary beds across the arteriolar tree (Jackson et al., 1987; Segal, 1994; Sprague et al., 1996; McCullough et al., 1997; Collins et al., 1998; Welsh and Segal, 1998; Dietrich et al., 2000).

Therefore, it has been further hypothesized that SO_2_-dependent ATP release from RBCs is initiated at the capillary level (Bergfeld and Forrester, 1992; Stein and Ellsworth, 1993; Ellsworth et al., 1995; McCullough et al., 1997; Stamler et al., 1997; Ellsworth, 2004; González-Alonso, 2012; Ellsworth et al., 2009; Ellis et al., 2012). Capillary beds are the most direct site for communicating tissue oxygen needs as the RBC membrane is in closest proximity to the endothelium compared to venules and arterioles (Ellis et al., 2012). This provides the shortest possible diffusion distance between capillary endothelium and RBCs resident in the capillary lumen allowing for ATP to rapidly diffuse and bind to endothelial purinergic receptors (Ellis et al., 2012). Capillary endothelial cells are indeed electrically coupled and can communicate electrical signals to the upstream arterioles (Bagher and Segal, 2011). In support of this hypothesis, several studies have shown that conducted signaling along capillaries can occur *in vivo* (Lamb et al., 2021; Collins et al., 1998; Dietrich, 1989; Dietrich and Tyml, 1992; Song and Tyml, 1993), and in the case of oxygen mediated responses, are dependent on connexin40 to conduct hyperpolarization to upstream arterioles (Kowalewska et al., 2024).

Intravital video microscopy (IVVM), which allows for monitoring and recording of real-time blood flow hemodynamics, has been frequently used to study blood flow in relation to O_2_ transport and sensing in microvascular networks (Dietrich and Tyml, 1992; Duling and Berne, 1970a; Potter et al., 1993; Welsh and Segal, 1998; Frisbee and Lombard, 2002; Frisbee et al., 2002). Using IVVM, O_2_-mediated blood flow can be studied within an intact system with multiple levels of vasoactive control during various experimental manipulations, such as altering tissue [O_2_] or locally micro-pipetting vasoactive stimuli onto an individual microvessel (Duling and Berne, 1970b; 1974; Jackson and Duling, 1983; Song and Tyml, 1993; Potter et al., 1993; Welsh and Segal, 1998; Frisbee and Lombard, 2002; Riemann et al., 2011). To confirm whether O_2_ sensing, and regulation occurs at the capillary level, related IVVM approaches were developed to gain additional insights into this vascular level (Ellis et al., 2012; Ghonaim et al., 2011; 2021; Sové et al., 2021; Russell McEvoy et al., 2021; Russell McEvoy et al., 2022). Specifically, a gas exchange chamber setup was used to alter the PO_2_ of an entire tissue surface, or in a microscale region of the rat extensor digitorum longus (EDL) muscle to stimulate microvascular networks and to observe their regulatory responses (Ellis et al., 2012; Ghonaim et al., 2011; 2021; Sové et al., 2021; Russell McEvoy et al., 2022; Kowalewska et al., 2024).

Gas-based micro-outlet devices fabricated to visualize highly localized responses at the microvascular level have shown promising results for the proposed SO_2_-dependent ATP-release mechanism of O_2_ regulation (Ellis et al., 2012; Ghonaim et al., 2011; 2013; 2021; Sové et al., 2021). Such devices aim to have the spatial specificity required to quantify individual capillary RBC hemodynamic responses to [O_2_] manipulations with limited interactions of nearby vasculature (Ghonaim et al., 2011; 2021; Sové et al., 2021). If the proposed mechanism is correct, this direct capillary perturbation helps assess if an ATP signal generated in individual capillary networks when O_2_ levels are depleted can initiate a conducted regulatory response upstream (Ghonaim et al., 2011; 2013). Micro-outlets with 100-μm diameters have been shown to effectively alter SO_2_ in single capillaries but not to provoke a blood flow response (Ghonaim et al., 2011). Although, larger micro-outlets that stimulated multiple capillaries obtained flow responses simultaneously with RBC SO_2_ changes (Ghonaim et al., 2021; Sové et al., 2021). However, mathematical models developed to aid in the interpretation for these devices did not consider the diffusive spread of O_2_ within the device’s exchange membrane, which was a novel insight in recent experimental work (Ghonaim et al., 2011; 2013; 2021; Sové et al., 2021). This limitation in the model underestimated the radial diffusion from our devices, meaning the area stimulated by [O_2_] perturbations was effectively larger than expected; resulting in poorer spatial specificity than was originally intended (Ghonaim et al., 2011; 2021; Sové et al., 2021). Therefore, determining the location for O_2_ sensing using fine spatial specificity of targeted O_2_ perturbations to micro-scale tissue regions remains incomplete (Ghonaim et al., 2011; 2013; 2021; Sové et al., 2016; Sové et al., 2021).

In this study, we aimed to develop and validate a thin-film micro-outlet device with improved spatial specificity to localize oxygen perturbations at the capillary level in skeletal muscle. An improvement from previous implementations, our device is designed to offer superior optical characteristics allowing for distortion-free visualization and analysis of capillaries being manipulated. This allows for capillaries directly overlying the micro-outlet, and at a distance from the outlet edge, to be recorded with IVVM simultaneously (Ghonaim et al., 2011; 2013; Sové et al., 2021). Our overarching hypothesis is that SO_2_-dependent ATP release from erythrocytes is a major mechanism of oxygen-mediated blood flow regulation that is initiated at the capillary level. We sought to test the spatial specificity of this response by fabricating circular micro-outlets of different diameters coupled to a microfluidic gas exchange chamber. We used micro-outlet devices to spatially target groups of capillaries in live muscle tissue with a range of [O_2_] perturbations with quantification of the resulting microhemodynamic responses. Furthermore, we validated our approach by applying an established mathematical model to predict PO_2_ conditions within the tissue and the oxygen permeable layers of our device, demonstrating a high degree of spatial specificity of imposed O_2_ perturbations. The current work redefines the critical scale for O_2_ sensing in skeletal muscle microcirculation, and provides further evidence for the role of capillaries in initiating highly localized O_2_-mediated blood flow regulatory responses.

## 2 Methods

### 2.1 Design and fabrication of the 3D printed gas exchange chamber

**FIGURE 1:**
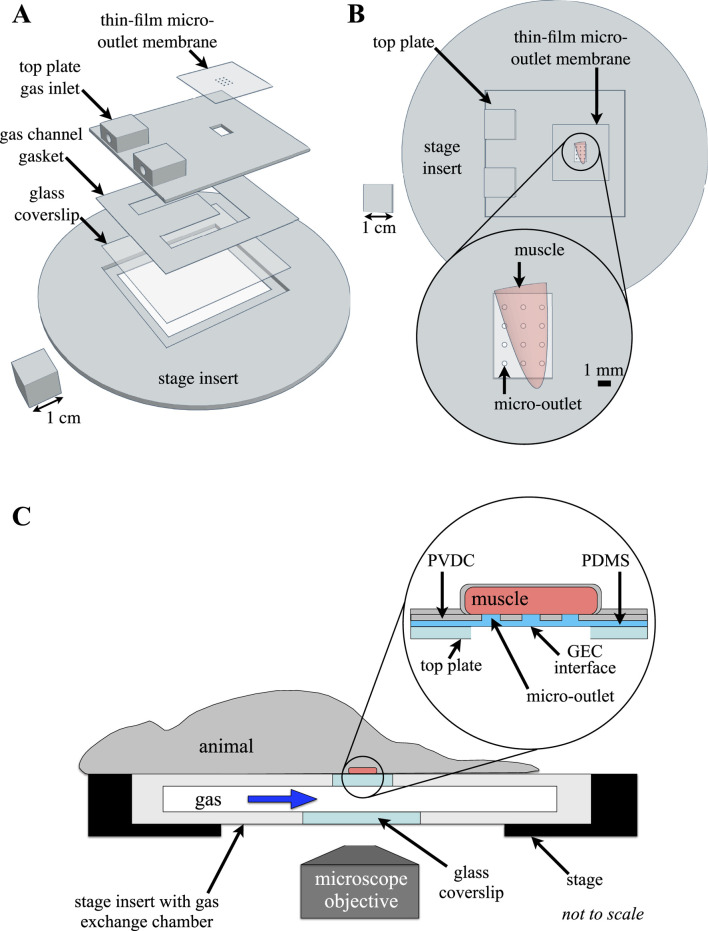
Experimental setup of the thin-film micro-outlet device and gas exchange chamber (GEC). The 3D printed GEC is fabricated to fit in the inverted microscope stage, with the extensor digitorum longus (EDL) muscle aligned directly over the thin-film micro-outlet device membrane. **(A)** shows an exploded view of the computer-aided design model of the GEC components illustrating device assembly. Top view of the assembled GEC with the EDL muscle overlying a 400 μm micro-outlet tessellation is shown in **(B)**. Micro-outlets were laser machined in polyvinylidene chloride (PVDC) barrier film which was coupled with polydimethylsiloxane (PDMS) to allow for diffusion between the GEC interface and the tissue overlying each micro-outlet. The top surface and sides of the muscle are isolated from room air by covering with a rectangle of gas-impermeable polyvinylidene chloride film and finally covered with a glass coverslip (not shown), ensuring the muscle is only affected by gases flowing through the chamber (**(C)** adapted from Sové et al., 2021, used under Creative Commons License).

The design of the gas exchange chamber (GEC) was adapted from previously reported computer-aided designs (Sové et al., 2021). Modifications of the GEC components used in this study were made using Tinkercad (Tinkercad.com, Accessed January 2021-September 2021). The GEC components consisted of a 110 mm diameter 3D printed stage insert base designed to fit the microscope stage cutout, with a micro gas channel formed between a 45 × 55 mm glass coverslip, a 0.15 mm thick 3D printed gas channel gasket, and an overlying 3D printed top plate (Figure 1). The top plate contained integral inlet and outlet ports to connect the GEC channel to the gas supply. The composite micro-outlet device membrane, described below, was mounted above a 4.5 × 6.5 mm exchange window fabricated in the top plate to allow coupling of the micro-outlet device with the GEC gas channel (Figure 1). The assembled device was sealed with adhesive vinyl sheets and connected by plastic tubing to a triple-inlet manifold supplied by three computer-controlled mass flow meters (SmartTrak100, Sierra Instruments, Monterey, CA, United States) for each gas channel (CO_2_, N_2_, O_2_), with a frequency response of <300 ms.

### 2.2 Micro-outlet device fabrication

#### 2.2.1 Fabrication of micro-outlets

Polyvinylidene chloride film (PVDC) (Asahi Wrap, Asahi Kasei, Tokyo, Japan) was used as the gas-impermeable layer in a composite membrane micro-outlet device. Micro-outlet holes were laser cut into PVDC film using high-precision laser cutting (Universal Laser Systems–Model # ILS12-150D) at 100% speed and 0.1% power on a single laser using high-precision cuts. Four micro-outlet tessellations designed for each outlet diameter were laser cut into gas impermeable PVDC film, with 1,000 μm of solid film separating each outlet cut. Each micro-outlet tessellation was patterned to fit within the perimeter of the 4.5 × 6.5 mm exchange window of the 3D printed GEC top plate (Figure 1). The tessellation pattern varied according to the diameter of micro-outlets used, with 200 μm outlets machined in a 5 × 4 (rows x columns) pattern, 400 μm and 600 μm outlets were machined in a 4 × 3 pattern, and 1,000 μm outlets were machined in a 3 × 2 pattern. To facilitate handling and subsequent spin coating of the PDMS membrane layer onto the PVDC film, each tessellation pattern was cut in the center of a 25 × 75 mm laser-cut piece of PVDC film. The micro-outlets cut into the PVDC film were inspected under a stereo microscope and thoroughly cleaned using 70% isopropyl solution and distilled water. A glass microscope slide was similarly cleaned and the 25 × 75 mm PVDC film containing the micro-outlet tessellation was carefully placed and smoothed onto a glass slide using 2-3 drops of distilled water and then left to dry in preparation for spin coating. The water droplets help to tightly adhere the PVDC film to the glass slide and prevent air bubbles from forming between the glass and film.

#### 2.2.2 Fabrication of composite gas exchange membranes

Gas impermeable laser-cut micro-outlet PVDC membranes were bonded to gas-permeable polydimethylsiloxane (PDMS) to create a composite device for spatially constrained gas exchange. A 10:1 (15 g:1.5 g) mixture of PDMS base and curing agent (Dow Corning, Midland, MI) was weighed and vigorously mixed for 2 min as directed by the manufacturer’s instructions. The PDMS mixture was degassed in a vacuum chamber three consecutive times for 10 min. Once degassed, the mixture was coated on top of a glass microscope slide with a micro-outlet film and spun coat for 30 s at 1,000 rpm. Following spin coating, the slide was transferred into a vacuum chamber for 10 min of degassing to ensure no bubbles were present in the PDMS covering the micro-outlet holes. The microscope slides and composite membrane were placed on a hot plate at 80°C for 20 min to initiate the curing of PDMS. After heating, the slide was covered with an inverted Petri dish to prevent dust particles from adhering to the device and left for 24 h to allow for complete curing of the PDMS. Once PDMS was cured on the PVDC film layer, the composite device was slowly peeled off the slide to ensure the delicate PDMS-filled micro-outlet holes remained intact. Once this was achieved, the composite device was placed directly over top of the 3D printed GEC top plate to align the tessellation pattern with the 3D printed window. The extra material of the composite membrane outside of the micro-outlet tessellation was trimmed, and the membrane was secured using double-sided tape and clear adhesive vinyl to ensure the composite micro-outlet device was fully sealed with the GEC.

### 2.3 Animal protocol

#### 2.3.1 Instrumentation and physiological monitoring

30 male Sprague-Dawley rats (159–194 g) were obtained from Charles River Laboratories and housed in Animal Care Facilities allowing them to acclimatize over a 5–7-day period before testing. Rats were fed Teklad 2018 (Envigo, Indianapolis, IND, United States) standard rodent chow. All animal protocols were approved by Memorial University Animal Care Committee.

To begin, animals were anesthetized using sodium pentobarbital (Euthanyl, Bimeda, Cambridge, ON, Canada) at 65 mg/kg through intraperitoneal injection. Following induction and prior to surgery, depth of anesthesia was assessed through palpebral reflex and the absence of withdrawal or reaction following firm toe pinch on the left foot. The animal was transferred to the surgical field and a rectal temperature probe was inserted to monitor body temperature. The animal’s core temperature was maintained between 36°C and 37°C throughout the experiment using a heating pad and/or a heat lamp as needed.

An incision was made between the clavicle and the jaw along the midline to allow for instrumentation as previously described (Tyml and Budreau, 1991; Fraser et al., 2012). Briefly, the left common carotid artery was blunt dissected and isolated for cannulation to allow for continuous monitoring and recording of blood pressure and heart rate (400a Blood Pressure Analyzer, Micro-Med, Louisville, KY, United States). The jugular vein was blunt dissected, isolated, and cannulated to deliver heparinized saline for fluid resuscitation (2 mL/kg/hr) via an infusion pump (PhD 2000, Harvard Apparatus, Holliston, MA, United States). The depth of anesthesia was assessed frequently by checking the animal’s blink response, monitoring heart rate variability, and mean arterial blood pressure changes. Supplemental anesthetic (22 mg/kg) was administered via the jugular cannula as required. Animals were tracheotomized and mechanically ventilated (Inspira ASV, Harvard Apparatus, Holliston, MA, United States) with a FiO_2_ of ∼30% O_2_ balanced with N_2_. Respiratory rate and volume were determined based on the animal’s weight per the manufacturer’s instructions. The right EDL, a muscle in the lower hindlimb of the rat, was blunt dissected and isolated from overlying connective tissue, as previously described (Fraser et al., 2012; Tyml and Budreau, 1991). The distal tendon of the EDL was cut, the muscle was lifted and carefully rinsed with warm saline before being reflected over the objective on the stage of an inverted microscope (Olympus IX73, Tokyo, Japan). The EDL was carefully positioned over the exchange window of the 3D-printed stage insert. To improve optical coherence, the muscle was gently compressed using a cover slip with two beads of vacuum grease (Dow Corning, Midland, MI, United States), and a microscope slide similarly prepared with parallel beads of vacuum grease. The EDL was covered with PVDC film for isolation from the outside environment (Saran, Dow Corning, MI, United States) and bathed in warm saline. The muscle surface overlying the microscope objective was interfaced with the GEC gas channel via the PDMS-filled micro-outlet holes within the composite device membrane. The animal was allowed to equilibrate on the microscope stage for 30 min after EDL positioning and setup were complete. Following equilibration and when the animal’s body temperature was between 36°C–37°C, and MAP was above 80 mmHg, an arterial blood sample was collected to measure blood gases (VetScan iSTAT, Abbott Point of Care Inc., Princeton, NJ, United States). The partial pressure of oxygen (PO_2_) and carbon dioxide (PCO_2_) was maintained at physiological levels, and the ventilation rate and volume were adjusted to maintain blood gases within the normative range.

### 2.4 Offline analysis using custom MATLAB software

Recorded digital video sequences were processed using a graphical user interface (GUI) driven custom program written in MATLAB (Mathworks, Natick, Mass, MA, United States). This processing software creates functional images for vessel selection and generates MP4 videos that help identify in-focus vessels for selection and analysis in the custom MATLAB software (Fraser et al., 2012; Ellis et al., 1990; 1992; 2012; Japee et al., 2004; Ghonaim et al., 2021). The analysis GUI measures capillary RBC hemodynamics and capillary RBC oxygen saturation (SO_2_) as has been described previously (Fraser et al., 2012; Ellis et al., 1990; 1992; 2012; Japee et al., 2004). Output from the analysis software underwent further structured quality control to exclude spurious values that can result from poor vessel delineation or out-of-focus segments.

### 2.5 Hemodynamic and oxygen saturation measurements

Time-dependent capillary RBC hemodynamic (velocity, supply rate, and hematocrit) and SO_2_ measurements were collected for capillaries during step-wise [O_2_] oscillations between 7% (1 min) → 12% (1 min) → 2% (1 min) → 7% (1 min), high [O_2_] challenges from 7% (1 min) → 12% (2 min), and low [O_2_] challenges from 7% (1 min) → 2% (2 min). For measurements obtained during 4-min [O_2_] oscillations, the mean for each data channel was calculated for the first minute at 7% [O_2_], and for the last 15 s for each subsequent 1-min period at 12%, 2%, and 7% [O_2_]. Similarly, for [O_2_] challenges, means were calculated for the first minute at 7% [O_2_], and for the last 15 s of the 2-min challenge period.

Capillary data for each micro-outlet size tested was sorted into four bins based on the location of analyzed capillary segments relative to the micro-outlet. The first bin included capillary segments ‘inside’ the micro-outlet, meaning the segments were directly overlying the micro-outlet surface and were resolved inside the perimeter of the laser machined hole in the PVDC layer. Data from capillary segments located ‘outside’ the micro-outlet was binned into three distance ranges: <100 μm, 100–200 μm, and >200 μm from the perimeter of the laser-machined hole in the PVDC layer. To facilitate distance measurements, the outline and mid-point of each analyzed capillary segment was annotated on functional images generated from the video sequences. Distances of each capillary segment’s mid-point to the perimeter of the micro-outlet hole were measured from the annotated functional images using ImageJ software (Rasband, W.S., ImageJ, U. S. National Institutes of Health, Bethesda, Maryland, United States, https://imagej.nih.gov/ij/, 1997–2018) and vessels were assigned to the appropriate distance bin. Hemodynamic measurements from capillaries crossing the micro-outlet with connected segments both ‘inside’ and ‘outside’, were only included in the ‘inside’ bin. Similarly, SO_2_ measurements from capillaries crossing the micro-outlet were only included in ‘outside’ bins if the segment was flowing towards the micro-outlet.

Capillary hemodynamic and SO_2_ measurements were sorted based on temporally paired measurements from [O_2_] oscillation and [O_2_] challenge data. Statistical comparisons were made for each imposed [O_2_]. For [O_2_] oscillations, normally distributed capillary data were paired across the four [O_2_] of the oscillation, and repeated measures one-way analysis of variance (ANOVA) with Holm-Šídák’s multiple comparisons test was used to identify significant differences between each [O_2_] condition. Non-normally distributed [O_2_] oscillation capillary data were grouped by [O_2_], and Friedman tests with Dunn’s multiple comparison post-test were used to identify significant differences between each [O_2_] condition of the oscillation. For [O_2_] challenges, normally distributed capillary data were paired across the two [O_2_] conditions, and a paired t-test was used to identify significant differences between the baseline period and end of the challenge. Non-normally distributed [O_2_] challenge capillary data were grouped by [O_2_], and a Wilcoxon test was used to identify significant differences between the baseline and end of the challenge. A *p* value of <0.05 was considered significant across all comparisons. All tests were completed using Prism 9 (GraphPad Prism Software, LLC, 9.2.0 (283)). Means and standard deviations are reported in the results section unless otherwise noted.

### 2.6 Mathematical model of tissue oxygenation

A mathematical model of oxygen diffusion was used to determine the expected PO_2_ within the tissue for the specific conditions imposed experimentally using the gas exchange chamber. Oxygen diffusion within the overlying muscle tissue and layers of the micro-outlet device were simulated using a finite element model similar to that described previously (Sové et al., 2021; Russell McEvoy et al., 2021). PO_2_ in the system was determined by numerically solving:

$$\nabla \cdot \left(D_{PDMS}\cdot k_{PDMS}\cdot \nabla p\right)=0,$$

in the PDMS layer,

$$\nabla \cdot \left({Dk}_{PVDC}\cdot k_{PVDC}\cdot \nabla p\right)=0,$$

in the PVDC layer, and

$$\nabla \cdot \left(D_{tissue}\cdot k_{tissue}\cdot \nabla p\right)+q\left(1-\frac{p}{p_{b}}\right)-M_{0}\frac{p}{p+p_{50}}=0,$$

in the tissue, where *M*_*0*_ is the maximal tissue oxygen consumption, *p*_*50*_ is the PO_2_ at which consumption is half *M*_*0*_, *p*_*b*_ is the effective capillary *PO*_*2*_ and *q* is the rate of oxygen transport from the capillaries into the tissue. *D* and *k* are the oxygen diffusivity and solubility in the medium denoted by the subscript. The model parameter values and associated references are listed in Supplementary Table S1. Fixed value boundary conditions for PO_2_ were prescribed at the interface of the system with the gas exchange chamber (see Figure 1), and zero flux boundary conditions were specified on all other boundaries of the volume. The governing equations were discretized using a finite volume approach and the resulting system of equations was solved iteratively by successive overrelaxation.

## 3 Results

### 3.1 Optical characteristics of thin-film micro-outlets

**FIGURE 2:**
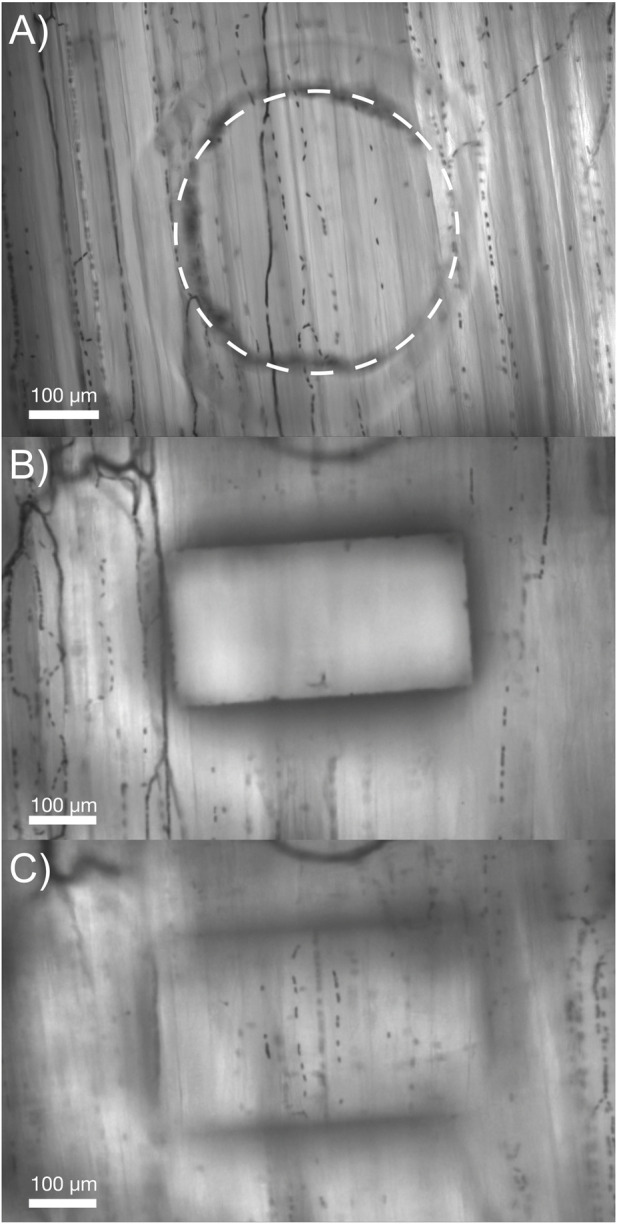
Comparison of the optical properties obtained in our micro-outlet device with a previous device used *in vivo*. **(A)** Image of our optically superior thin-film 400 μm micro-outlet device *in vivo*. **(B,C)** Images of a previous micro-outlet device demonstrating the inability to simultaneously visualize in-focus vessels overlying, and adjacent to the outlet. Images in **(B,C)** are courtesy of Dr. Chris Ellis’ Lab at Western University, collected as part of the study by Sové et al., 2021.

The thin-film micro-outlet devices fabricated for the current study produced superior optical clarity compared to previous devices using glass substrates. Our current approach of spin coating PDMS onto PVDC films containing laser machined micro-outlets allows for capillaries in the same focal plane, both overlying the micro-outlet and at a distance, to be clearly resolved simultaneously (Figure 2).

### 3.2 Systemic physiological measurements

Systemic physiological measurements were taken for each animal and means are reported for each group according to micro-outlet diameter size as shown in Table 1. Similarly, blood gas samples were collected for each animal and are displayed in Table 2 grouped based on corresponding micro-outlet diameter sizes.

**TABLE 1:** Mean and standard deviation of systemic animal data for each experimental group based on micro-outlet diameter size.

	200 μm outlets (N = 6)	400 μm outlets (N = 6)	600 μm outlets (N = 11)	1000 μm outlets (N = 7)
Animal Weight (g)	179.5 ± 10.7	178.5 ± 7.7	176.7 ± 7.3	187.1 ± 4.3
Mean arterial pressure (mmHg)	94.6 ± 6.0	94.2 ± 8.8	94.54 ± 5.4	96.93 ± 5.5
Systolic blood pressure (mmHg)	99.0 ± 6.7	99.1 ± 9.0	103.0 ± 7.8	111.1 ± 5.6
Diastolic blood pressure (mmHg)	87.9 ± 5.4	87.2 ± 8.9	84.0 ± 6.8	82.0 ± 5.4
Heart rate (beats/min)	411.9 ± 21.2	403.0 ± 24.8	394.6 ± 20.7	399.2 ± 16.8
Respiratory Rate (breaths/min)	83.5 ± 1.4	83.3 ± 1.2	83.8 ± 0.9	82.4 ± 0.5
Respiratory volume (cc)	1.20 ± 0.07	1.18 ± 0.05	1.17 ± 0.06	1.24 ± 0.03

**TABLE 2:** Mean and standard deviation of animal blood gas data for each experimental group based on micro-outlet diameter size used.

	200 μm outlets (N = 6)	400 μm outlets (N = 6)	600 μm outlets (N = 11)	1000 μm outlets (N = 7)
pH	7.40 ± 0.03	7.42 ± 0.02	7.43 ± 0.05	7.42 ± 0.04
PCO_2_ (mmHg)	50.0 ± 3.8	45.8 ± 3.5	46.4 ± 6.6	48.3 ± 5.8
PO_2_ (mmHg)	113.8 ± 15.4	102.5 ± 9.2	114.0 ± 17.7	120.1 ± 10.3
BEecf (mmol/L)	6.3 ± 1.2	5.5 ± 1.9	5.8 ± 2.3	6.9 ± 2.2
HCO_3_ (mmol/L)	29.4 ± 4.4	29.8 ± 1.8	30.1 ± 2.1	31.3 ± 2.4
SaO_2_ (%)	98.0 ± 0.9	97.8 ± 0.8	98.2 ± 1.3	98.7 ± 0.5
Lac (mmol/L)	0.87 ± 0.40	0.87 ± 0.21	1.01 ± 0.68	1.01 ± 0.43

Note: PCO_2_: partial pressure of carbon dioxide; PO_2_: partial pressure of oxygen; BEecf: base excess in the extracellular fluid compartment concentration; HCO_3_: bicarbonate concentration; TCO_2_: total carbon dioxide; SaO_2_: arterial oxygen saturation; Lac: lactate concentration.

### 3.3 400 µm micro-outlet oxygen oscillation data

**FIGURE 3:**
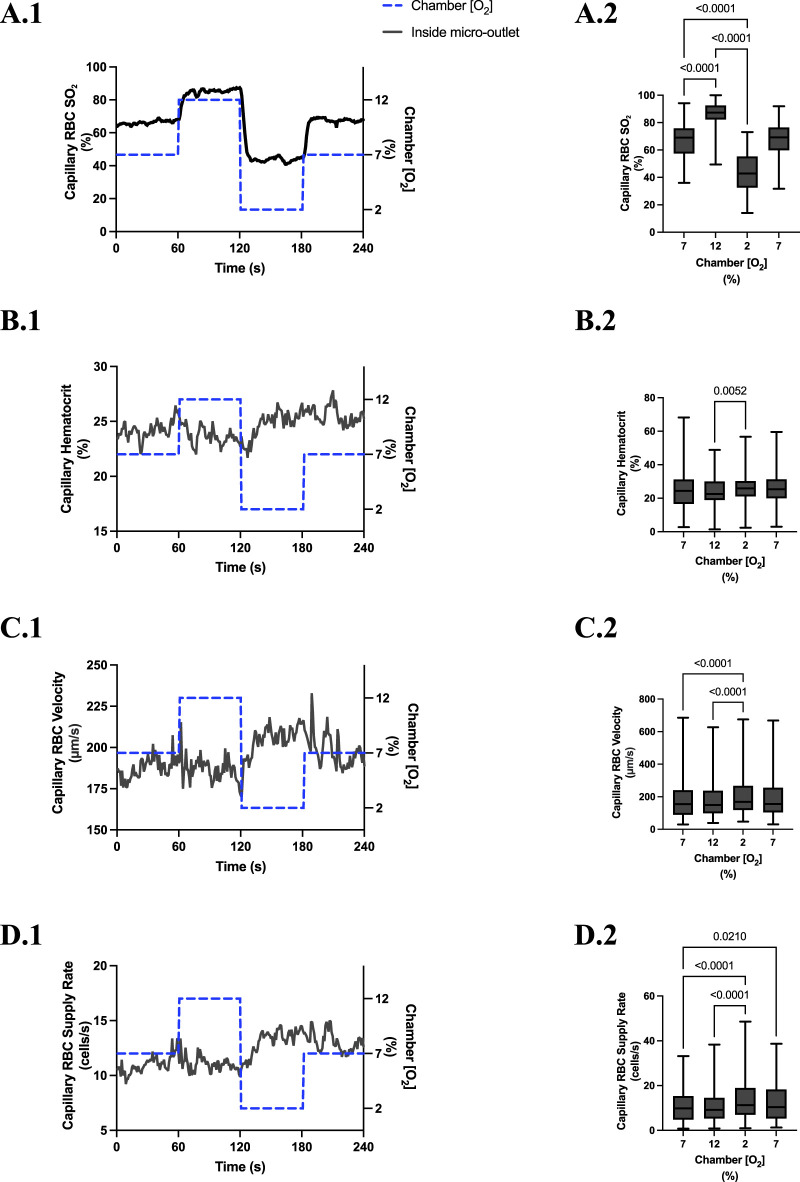
Capillary red blood cell (RBC) oxygen saturation (SO_2_) and hemodynamic responses to oxygen concentration ([O_2_]) oscillations for vessels directly overlying 400 µm micro-outlets. The micro-outlet gas exchange device imposed a 4-min [O_2_] oscillation consisting of 1-min [O_2_] perturbations at 7%, 12%, 2%, and 7%, respectively. Time series plots show the mean capillary RBC (SO_2_) **(A.1)**, hematocrit **(B.1)**, velocity **(C.1)**, and supply rate **(D.1)** in capillaries overlying the 400 µm micro-outlet edge across the 4-min [O_2_] oscillation (7% (1 min) *→*12% (1 min) *→*2% (1 min) *→*7% (1 min)). Comparisons between the baseline 7% period and the oscillation perturbations for capillary RBC SO_2_ **(A.2)**, hematocrit **(B.2)**, velocity **(C.2)** and supply rate **(D.2)** were made using mean values taken from the entire first minute at baseline 7% [O_2_] and the last 15 s at 12%, 2%, and 7% (n = 86 capillaries). *p* values indicated in the figure with a *p* < 0.05 are considered significant. Box and whisker plots show minimum, median, maximum, and associated quartiles.

**FIGURE 4:**
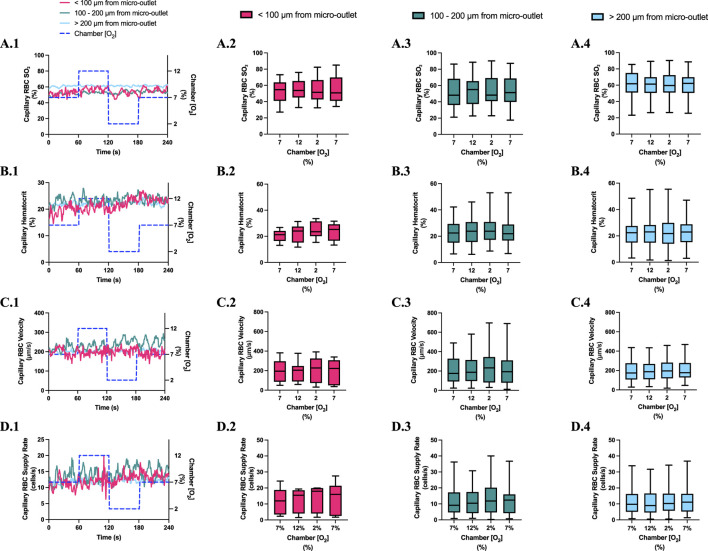
Capillary red blood cell (RBC) oxygen saturation (SO_2_) and hemodynamic responses to oxygen concentration ([O_2_]) oscillations for vessels at various distances outside the 400 µm micro-outlets. **(A.1–D.1)** represent time series plots for mean capillary RBC (SO_2_), hematocrit, velocity, and supply rate, respectively for capillaries outside the micro-outlet edge at varying distances across the 4-min O_2_ oscillations (7→12→2→7%). The micro-outlet gas exchange device imposed 4-min [O_2_] oscillations consisting of 1-min [O_2_] perturbations at 7%, 12%, 2%, and 7%, respectively. **(A.2–A.4)** represents mean capillary RBC SO_2_ of capillaries within 100 µm from the micro-outlet edge (n = 8 capillaries), between 100–200 µm from the outlet edge (n = 34 capillaries), and greater than 200 µm from the outlet edge (n = 71 capillaries), respectively. **(B.2–B.4)** represents mean capillary hematocrit of capillaries within 100 µm from the micro-outlet edge (n = 8 capillaries), between 100–200 µm from the outlet edge (n = 35 capillaries), and greater than 200 µm from the outlet edge (n = 73 capillaries), respectively. **(C.2–C.4)** represents mean capillary RBC velocity of capillaries within 100 µm from the micro-outlet edge (n = 8 capillaries), between 100–200 µm from the outlet edge (n = 35 capillaries), and greater than 200 µm from the outlet edge (n = 73 capillaries), respectively. **(D.2–D.4)** represents mean capillary RBC supply rate of capillaries within 100 µm from the micro-outlet edge (n = 8 capillaries), between 100–200 µm from the outlet edge (n = 35 capillaries), and greater than 200 µm from the outlet edge (n = 73 capillaries), respectively. Box and whisker plots show minimum, median, maximum, and associated quartiles.

[O_2_] oscillations imposed on capillaries directly overlying 400 μm micro-outlets caused significant changes in capillary RBC SO_2_ at 12% GEC [O_2_], 85.9% ± 9.6%, and 2% GEC [O_2_], 43.2% ± 14.8%, compared to baseline 7% GEC [O_2_], 66.2% ± 13.2% (p < 0.0001) (Figure 3). Imposed [O_2_] oscillations caused no significant changes in SO_2_ in capillaries outside the micro-outlet edge (Figure 4).

[O_2_] oscillations imposed on capillaries directly overlying 400 μm micro-outlets caused significant changes in capillary hematocrit in capillaries overlying the outlet at 12% GEC [O_2_], 23.5% ± 10.1% compared to 2% GEC [O_2_], 25.4% ± 9.7% (*p* = 0.005) (Figure 3), but not in capillaries outside the outlet (Figure 4). [O_2_] oscillations imposed on capillaries directly overlying 400 μm micro-outlets caused significant changes in capillary RBC velocity at 2% GEC [O_2_], 211.5 ± 130.2 µm/s, compared to baseline 7% GEC [O_2_], 187.4 ± 134.2 µm/s and at 12% GEC [O_2_], 185.1 ± 122.1 µm/s compared to 2% GEC [O_2_], 211.5 ± 130.2 µm/s (p < 0.0001) (Figure 3). [O_2_] oscillations imposed on capillaries directly overlying 400 μm micro-outlets did not cause significant changes in capillary RBC velocity in vessels outside the micro-outlet (Figure 4).

[O_2_] oscillations imposed on capillaries directly overlying 400 μm micro-outlets caused significant changes in capillary RBC supply rate at 2% GEC [O_2_], 13.8 ± 9.7 cells/s, and 7% GEC [O_2_], 12.8 ± 9.3 cells/s compared to baseline 7% GEC [O_2_] 11.0 ± 7.7 cells/s and at 12% GEC [O_2_], 10.8 ± 8.0 cells/s, compared to 2% GEC [O_2_], 13.8 ± 9.7 cells/s (p < 0.0210) (Figure 3). [O_2_] oscillations using 400 μm micro-outlets did not cause significant changes in capillary RBC supply rate in vessels outside the micro-outlet (Figure 4).

### 3.4 400 µm micro-outlet oxygen challenge data

**FIGURE 5:**
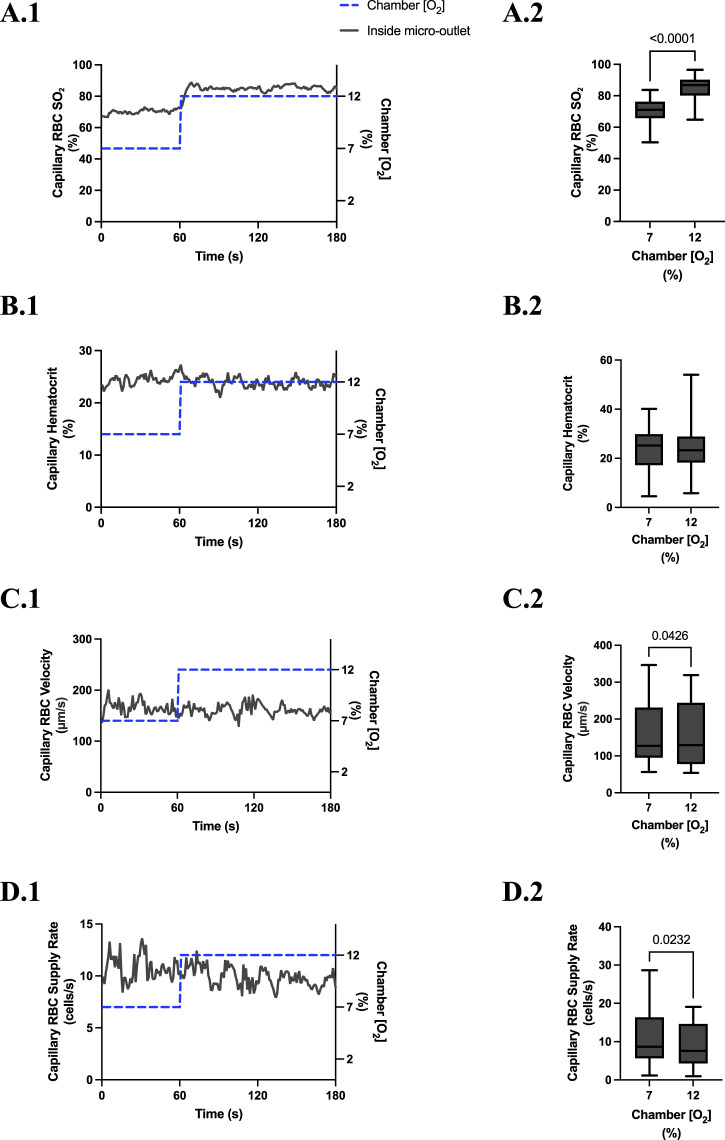
Capillary red blood cell (RBC) oxygen saturation (SO_2_) and hemodynamic responses in capillaries directly overlying the 400 µm micro-outlet edge in response to high oxygen concentration ([O_2_]) challenges. [O_2_] challenges began with 1-min baseline [O_2_] at 7% followed by 2 min at 12%. Time series plots are displayed in **(A.1–D.1)** for mean capillary RBC SO_2_ **(A.1)**, hematocrit **(B.1)**, velocity **(C.1)**, and supply rate **(D.1)**, for capillaries overlying the micro-outlet during low [O_2_] challenges. For **(A.2–D.2)**, the average was taken from the entire first minute at 7% and the last 15 s at 12% [O_2_]. **(A.2–D.2)** represent mean RBC SO_2_ (n = 28 capillaries), hematocrit, velocity, and supply rate (n = 28 capillaries), respectively, for capillaries directly overlying the 200 µm micro-outlet. *p* values indicated in the figure with a *p* < 0.05 are considered significant. Box and whisker plots show minimum, median, maximum, and associated quartiles.

**FIGURE 6:**
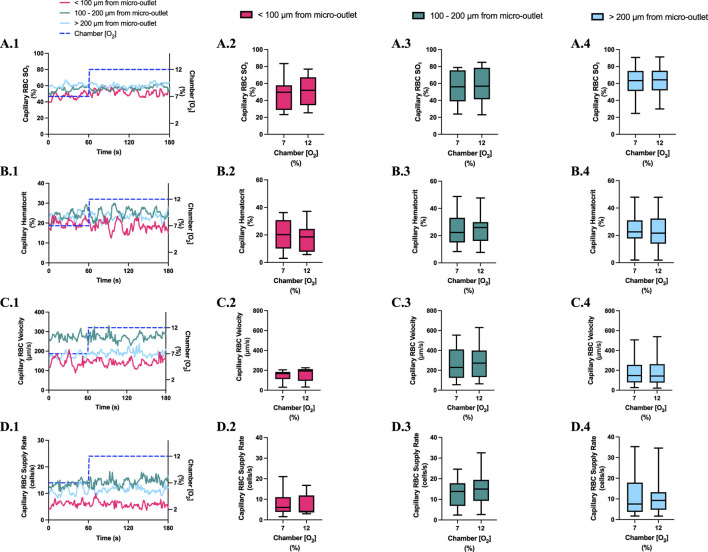
Capillary red blood cell (RBC) oxygen saturation (SO_2_) and hemodynamic responses in capillaries at various distances outside the 400 µm micro-outlet edge in response to high oxygen concentration ([O_2_]) challenges. **(A.1–D.1)** represent time series plots for mean capillary RBC SO_2_, hematocrit, velocity, and supply rate, respectively, for capillaries outside the micro-outlet edge at varying distances during low (7→12%) [O_2_] challenges. [O_2_] challenges consisted of 1 min at a baseline [O_2_] of 7% followed by 2 min at 12%. The average was taken from the entire first minute at 7% and the last 15 s at a 12% [O_2_]. **(A.2–A.4)** represents mean capillary RBC SO_2_ of capillaries within 100 µm from the micro-outlet edge (n = 8 capillaries), between 100–200 µm from the outlet edge (n = 14 capillaries), and greater than 200 µm from the outlet edge (n = 25 capillaries), respectively. **(B.2–B.4)** represents mean capillary hematocrit of capillaries within 100 µm from the micro-outlet edge (n = 9 capillaries), between 100–200 µm from the outlet edge (n = 14 capillaries), and greater than 200 µm from the outlet edge (n = 26 capillaries), respectively. Panel **(C.2–C.4)** represents mean capillary RBC velocity of capillaries within 100 µm from the micro-outlet edge (n = 9 capillaries), between 100–200 µm from the outlet edge (n = 14 capillaries), and greater than 200 µm from the outlet edge (n = 26 capillaries), respectively. **(D.2–D.4)** represents mean capillary RBC supply rate of capillaries within 100 µm from the micro-outlet edge (n = 9 capillaries), between 100–200 µm from the outlet edge (n = 14 capillaries), and greater than 200 µm from the outlet edge (n = 26 capillaries), respectively. p values indicated in the figure with a *p* < 0.05 are considered significant. Box and whisker plots show minimum, median, maximum, and associated quartiles.

**FIGURE 7:**
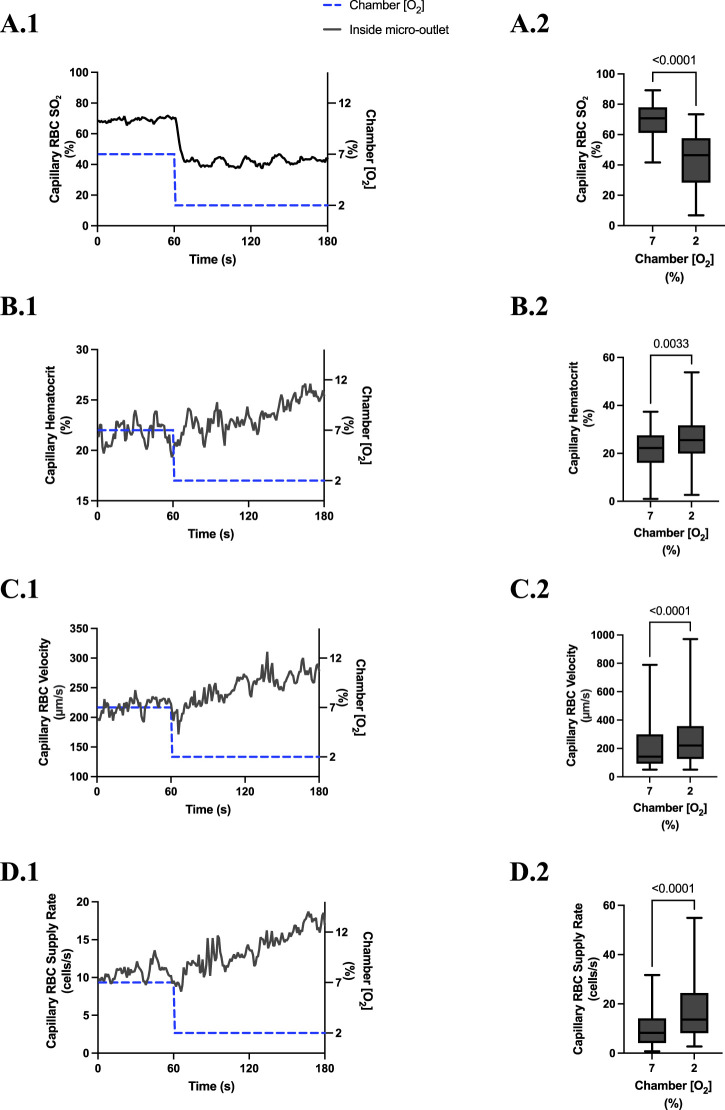
Capillary red blood cell (RBC) oxygen saturation (SO_2_) and hemodynamic responses in capillaries directly overlying the 400 µm micro-outlet edge in response to low oxygen concentration ([O_2_]) challenges. [O_2_] challenges began with 1-min baseline [O_2_] at 7% followed by 2 min at 2%. Time series plots are displayed in **(A.1–D.1)** for mean capillary RBC SO_2_ **(A.1)**, hematocrit **(B.1)**, velocity **(C.1)**, and supply rate **(D.1)**, for capillaries overlying the micro-outlet during low [O_2_] challenges. For **(A.2–D.2)**, the average was taken from the entire first minute at 7% and the last 15 s at 2% [O_2_]. **(A.2–D.2)** represent mean RBC SO_2_ (n = 36 capillaries), hematocrit, velocity, and supply rate (n = 36 capillaries), respectively, for capillaries directly overlying the 400 µm micro-outlet. p values indicated in the figure with a p < 0.05 are considered significant. Box and whisker plots show minimum, median, maximum, and associated quartiles.

**FIGURE 8:**
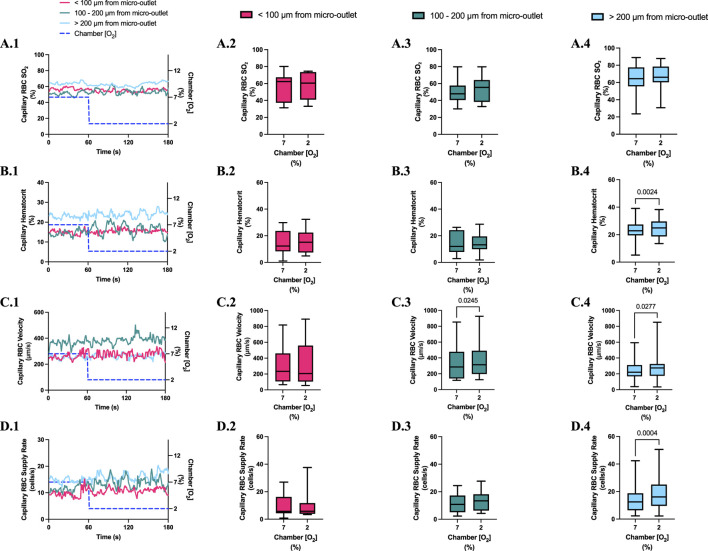
Capillary red blood cell (RBC) oxygen saturation (SO_2_) and hemodynamic responses in capillaries at various distances outside the 400 µm micro-outlet edge in response to low oxygen concentration ([O_2_]) challenges. **(A.1–D.1)** represent time series plots for mean capillary RBC SO_2_, hematocrit, velocity, and supply rate, respectively, for capillaries outside the micro-outlet edge at varying distances during low (7→2%) [O_2_] challenges. [O_2_] challenges consisted of 1 min at a baseline [O_2_] of 7% followed by 2 min at 2%. The average was taken from the entire first minute at 7% and the last 15 s at a 2% [O_2_]. **(A.2–A.4)** represents mean capillary RBC SO_2_ of capillaries within 100 µm from the micro-outlet edge (n = 7 capillaries), between 100–200 µm from the outlet edge (n = 11 capillaries), and greater than 200 µm from the outlet edge (n = 17 capillaries), respectively. **(B.2–B.4)** represents mean capillary hematocrit of capillaries within 100 µm from the micro-outlet edge (n = 15 capillaries), between 100–200 µm from the outlet edge (n = 14 capillaries), and greater than 200 µm from the outlet edge (n = 23 capillaries), respectively. **(C.2–C.4)** represents mean capillary RBC velocity of capillaries within 100 µm from the micro-outlet edge (n = 15 capillaries), between 100–200 µm from the outlet edge (n = 14 capillaries), and greater than 200 µm from the outlet edge (n = 23 capillaries), respectively. **(D.2–D.4)** represents mean capillary RBC supply rate of capillaries within 100 µm from the micro-outlet edge (n = 15 capillaries), between 100–200 µm from the outlet edge (n = 14 capillaries), and greater than 200 µm from the outlet edge (n = 23 capillaries), respectively. p values indicated in the figure with a *p* < 0.05 are considered significant. Box and whisker plots show minimum, median, maximum, and associated quartiles.

[O_2_] challenges imposed on capillaries directly overlying 400 μm micro-outlets caused significant changes in capillary RBC SO_2_ at 12% GEC [O_2_], 84.7% ± 8.5%, compared to baseline 7% GEC [O_2_], 70.1% ± 8.5% (p < 0.0001) (Figure 5). [O_2_] challenges imposed on capillaries directly overlying 400 μm micro-outlets caused significant changes in RBC SO_2_ at 2% GEC [O_2_], 43.1% ± 16.8%, compared to baseline 7% GEC [O_2_], 69.1% ± 12.1% (p < 0.0001) (Figure 7). Both high and low [O_2_] challenges imposed on capillaries caused no significant change in mean RBC SO_2_ for capillaries outside the micro-outlet edge (Figures 6, 8).

[O_2_] challenges imposed on capillaries directly overlying 400 μm micro-outlets caused a significant change in capillary hematocrit at 2% GEC [O_2_], 25.7% ± 9.4%, compared to baseline 7% GEC [O_2_], 21.8% ± 9.4% (p < 0.0001) but not at 12% GEC [O_2_] (Figures 5, 7). High [O_2_] challenges did not cause significant changes in capillary hematocrit for vessels at various distances from the outlet edge but there was a significant change at 2% GEC [O_2_], 25.4% ± 7.0%, compared to baseline 7% GEC [O_2_], 22.8% ± 7.6% for vessels greater than 200 μm away during low [O_2_] challenges (*p* = 0.0024) (Figures 6, 8).

[O_2_] challenges imposed on capillaries directly overlying 400 μm micro-outlets caused significant changes in capillary RBC velocity at 12% GEC [O_2_], 157.0 ± 84.4 µm/s, compared to baseline 7% GEC [O_2_], 165.0 ± 89.9 µm/s (*p* = 0.0426) (Figure 5). [O_2_] challenges imposed on capillaries directly overlying 400 μm micro-outlets caused significant changes in capillary RBC velocity at 2% GEC [O_2_], 274.4 ± 210.4 µm/s, compared to baseline 7% GEC [O_2_], 218.6 ± 189.0 µm/s (*p* < 0.0001) (Figure 7). Low [O_2_] challenges caused a significant increase in capillary RBC velocity in vessels between 100 and 200 μm away from the outlet edge at 2% GEC [O_2_], 385.9 ± 237.4 µm/s, compared to baseline 7% GEC [O_2_], 355.6 ± 244.1 µm/s (*p* = 0.0245) (Figure 8). Vessels greater than 200 μm away from the outlet edge experienced significant changes in capillary RBC velocity at 2% GEC [O_2_], 282.0 ± 178.9 µm/s, compared to baseline 7% GEC [O2], 253.9 ± 153.0 µm/s (*p* = 0.0277) (Figure 8).

High [O_2_] challenges imposed on capillaries directly overlying 400 μm micro-outlets caused significant changes in capillary RBC supply rate at 12% GEC [O_2_], 9.5 ± 5.7 cells/s, compared to baseline 7% GEC [O_2_], 10.7 ± 7.2 cells/s (*p* = 0.0232) (Figure 5). Low [O_2_] challenges imposed on capillaries directly overlying 400 μm micro-outlets caused significant changes in capillary RBC supply rate at 2% GEC [O_2_], 17.7 ± 13.4 cells/s, compared to baseline 7% GEC [O_2_], 10.7 ± 8.1 cells/s (*p* < 0.0001) (Figure 7). [O_2_] challenges imposed on capillaries directly overlying 400 μm micro-outlets caused significant changes in capillary RBC supply rate in vessels greater than 200 μm from the outlet edge at 2% GEC [O_2_], 18.4 ± 12.2 cells/s, compared to a baseline 7% GEC [O_2_], 14.7 ± 10.5 cells/s (*p* = 0.0004) (Figure 8). No other outlet vessels had significant changes in capillary RBC supply rate during high and low [O_2_] challenges.

**FIGURE 9:**
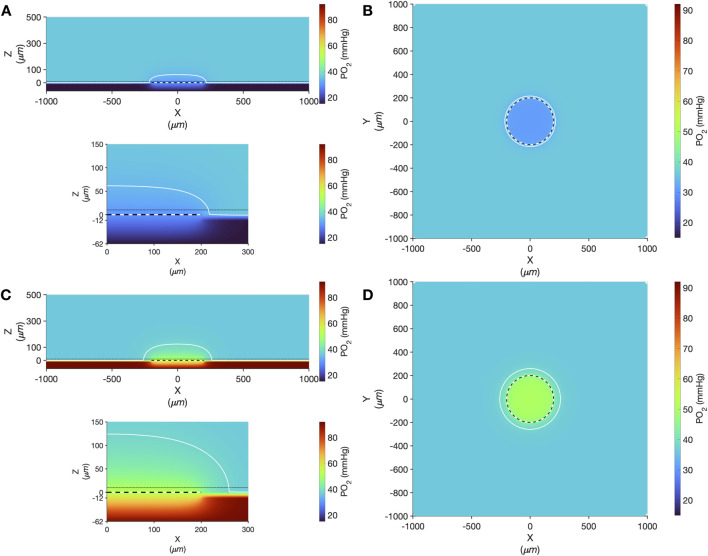
Oxygen transport simulation predicting tissue PO_2_ resulting from diffusional exchange between the tissue and gas exchange chamber via a 400 µm diameter thin-film micro-outlet device. In all panels the micro-outlet surface is indicated by the white and black dashed line. Simulation results for low [O_2_] challenges at 2% [O_2_] in the chamber are shown at the centre of the outlet in the XZ plane **(A)**. The dotted-dashed line in **(A)** indicates the location of the XY plane shown in panel B that is 10 µm into the tissue volume. Background tissue PO_2_ within the volume at a distance from the micro-outlet is 36.6 mmHg, with the iso-line in **(A–D)** delineating the volume of tissue overlying the micro-outlet that experiences a difference up to ±2 mmHg during the [O_2_] challenges. Simulation results for high [O_2_] challenges at 12% [O_2_] in the chamber are shown at the middle of the outlet in the XZ plane **(C)** and at a depth of 10 µm into the tissue volume in the XY plane **(D)**. The lower panel in **(A,C)** show a magnified view of the interface between the micro-outlet device and the overlying tissue.

Computer simulation results showing the steady state PO_2_ within the tissue and micro-outlet device for high (12%) and low (2%) [O_2_] challenges are presented in Figure 9. Simulations predict that the volume perturbed by the 400 μm diameter micro-outlet are highly constrained to the tissue region directly overlying the micro-outlet-to-tissue interface. Importantly, the model predicts that the PVDC barrier layer in the micro-outlet device presents considerable resistance to oxygen diffusion, effectively constraining gas flux to the PDMS surface of the micro-outlet. Simulations of steady state conditions while the gas exchange chamber [O_2_] is held at 2% predicted that PO_2_ within the tissue volume would be decreased by >2 mmHg up to a radial distance of 17.0 μm beyond the micro-outlet edge, and up to 61.0 μm vertically into the tissue volume in the Z-axis along the center of the circular micro-outlet. At 2% [O_2_], tissue PO_2_ at the center of the micro-outlet-tissue interface was predicted to be 29.9 mmHg, and 30.8 mmHg directly above the micro-outlet 10 μm into the tissue volume (Figure 9). Simulations for high (12%) [O_2_] within the GEC predicted an increase in tissue PO_2_ of >2 mmHg up to a radial distance of 59.0 μm beyond the micro-outlet edge, and a depth into the tissue volume up to 125.0 μm (Figure 9). At 12% [O_2_], tissue PO_2_ at the center of the micro-outlet-tissue interface was predicted to be 52.7 mmHg, and 50.3 mmHg directly above the micro-outlet 10 μm into the tissue volume (Figure 9).

### 3.5 200 µm micro-outlet oxygen challenge data

**FIGURE 10:**
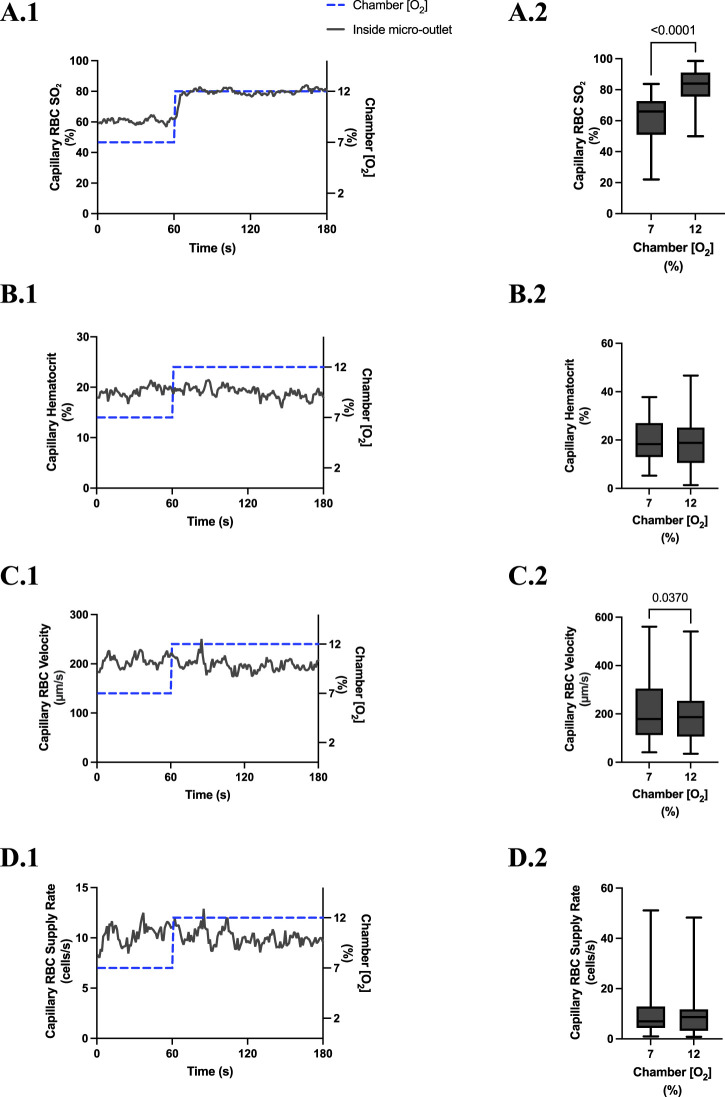
Capillary red blood cell (RBC) oxygen saturation (SO_2_) and hemodynamic responses in capillaries directly overlying the 200 µm micro-outlet edge in response to high oxygen concentration ([O_2_]) challenges. [O_2_] challenges began with 1-min baseline [O_2_] at 7% followed by 2 min at 12%. Time series plots are displayed in **(A.1–D.1)** for mean capillary RBC SO_2_ **(A.1)**, hematocrit **(B.1)**, velocity **(C.1)**, and supply rate **(D.1)**, for capillaries overlying the micro-outlet during low [O_2_] challenges. For **(A.2–D.2)**, the average was taken from the entire first minute at 7% and the last 15 s at 12% [O_2_]. **(A.2–D.2)** represent mean RBC SO_2_ (n = 44 capillaries), hematocrit, velocity, and supply rate (n = 42 capillaries), respectively, for capillaries directly overlying the 200 µm micro-outlet. *p* values indicated in the figure with a *p* < 0.05 are considered significant. Box and whisker plots show minimum, median, maximum, and associated quartiles.

High [O_2_] challenges imposed on capillaries directly overlying 200 μm micro-outlets caused significant elevation in capillary RBC SO_2_ at 12% GEC [O_2_], 81.3% ± 13.0%, compared to baseline 7% GEC [O_2_], 60.6% ± 16.9% (*p* < 0.0001) (Figure 10). Low [O_2_] challenges imposed on capillaries directly overlying 200 μm micro-outlets caused significant decrease in capillary RBC SO_2_ at 2% GEC [O_2_], 43.7% ± 18.7%, compared to baseline 7% GEC [O_2_], 65.3% ± 13.0% (*p* < 0.0001) (Figure 13).

**FIGURE 11:**
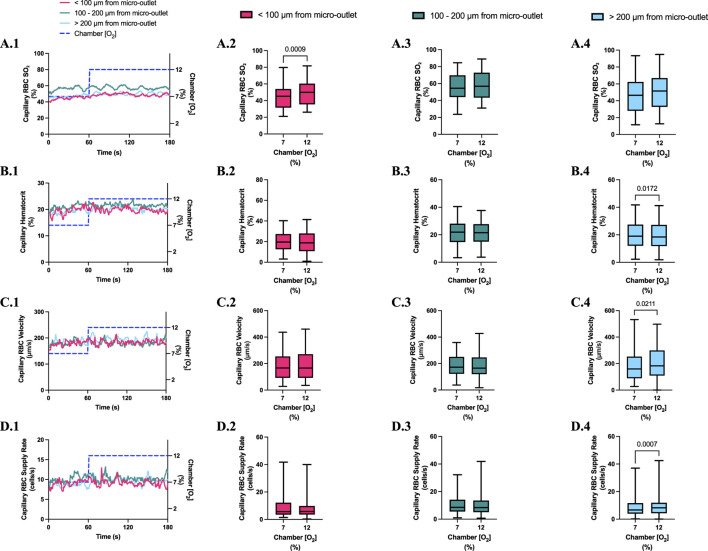
Capillary red blood cell (RBC) oxygen saturation (SO_2_) and hemodynamic responses in capillaries at various distances outside the 200 µm micro-outlet edge in response to high oxygen concentration ([O_2_]) challenges. **(A.1–D.1)** represent time series plots for mean capillary RBC SO_2_, hematocrit, velocity, and supply rate, respectively, for capillaries outside the micro-outlet edge at varying distances during high (7→12%) [O_2_] challenges. [O_2_] challenges consisted of 1 min at a baseline [O_2_] of 7% followed by 2 min at 12%. Means were calculated from the entire first minute at 7% and the last 15 s at a 12% [O_2_]. **(A.2–A.4)** represents mean capillary RBC SO_2_ of capillaries within 100 µm from the micro-outlet edge (n = 37 capillaries), between 100–200 µm from the outlet edge (n = 38 capillaries), and greater than 200 µm from the outlet edge (n = 48 capillaries), respectively. **(B.2–B.4)** represents mean capillary hematocrit of capillaries within 100 µm from the micro-outlet edge (n = 31 capillaries), between 100–200 µm from the outlet edge (n = 54 capillaries), and greater than 200 µm from the outlet edge (n = 68 capillaries), respectively. **(C.2–C.4)** represents mean capillary RBC velocity of capillaries within 100 µm from the micro-outlet edge (n = 31 capillaries), between 100–200 µm from the outlet edge (n = 54 capillaries), and greater than 200 µm from the outlet edge (n = 68 capillaries), respectively. **(D.2–D.4)** represents mean capillary RBC supply rate of capillaries within 100 µm from the micro-outlet edge (n = 31 capillaries), between 100–200 µm from the outlet edge (n = 54 capillaries), and greater than 200 µm from the outlet edge (n = 68 capillaries), respectively. p values indicated in the figure with a *p* < 0.05 are considered significant. Box and whisker plots show minimum, median, maximum, and associated quartiles.

**FIGURE 12:**
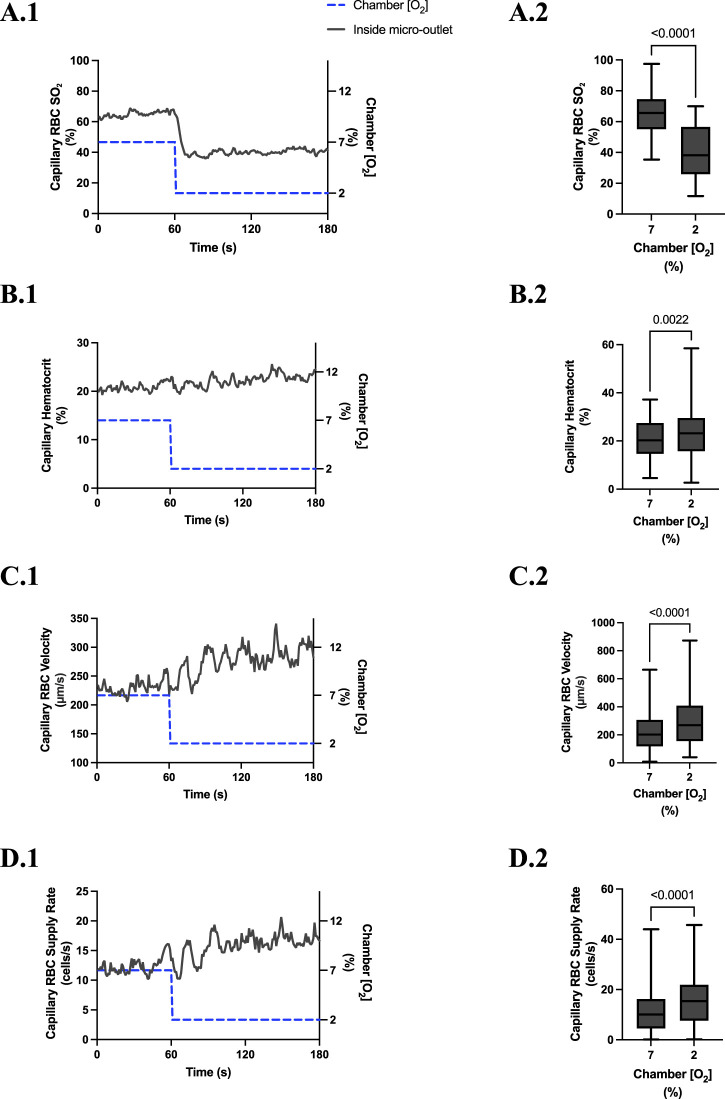
Capillary red blood cell (RBC) oxygen saturation (SO_2_) and hemodynamic responses in capillaries directly overlying the 200 µm micro-outlet edge in response to low oxygen concentration ([O_2_]) challenges. [O_2_] challenges began with 1-min baseline [O_2_] at 7% followed by 2 min at 2%. Time series plots are displayed in **(A.1–D.1)** for mean capillary RBC SO_2_ **(A.1)**, hematocrit **(B.1)**, velocity **(C.1)**, and supply rate **(D.1)**, for capillaries overlying the micro-outlet during low [O_2_] challenges. For **(A.2–D.2)**, the average was taken from the entire first minute at 7% and the last 15 s at 2% [O_2_]. **(A.2–D.2)** represent mean RBC SO_2_ (n = 43 capillaries), hematocrit, velocity, and supply rate (n = 53 capillaries), respectively, for capillaries directly overlying the 200 µm micro-outlet. *p* values indicated in the figure with a *p* < 0.05 are considered significant. Box and whisker plots show minimum, median, maximum, and associated quartiles.

High [O_2_] challenges imposed on capillaries directly overlying 200 μm micro-outlets caused significant increase in capillary RBC SO_2_ at 12% GEC [O_2_], 49.2% ± 15.6%, compared to baseline 7% GEC [O_2_], 44.5% ± 15.4% in capillaries less than 100 μm away from the outlet edge (*p* = 0.0009) (Figure 11). There were no significant increases or decreases in capillary RBC SO_2_ at 2% GEC [O_2_], compared to baseline 7% GEC [O_2_], in capillaries outside the micro-outlet at any distance from edge (Figure 12).

**FIGURE 13:**
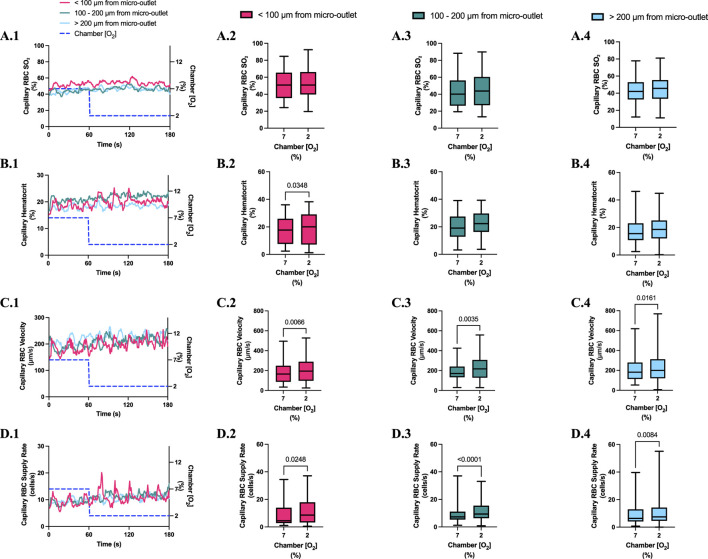
Capillary red blood cell (RBC) oxygen saturation (SO_2_) and hemodynamic responses in capillaries at various distances outside the 200 µm micro-outlet edge in response to low oxygen concentration ([O_2_]) challenges. **(A.1–D.1)** represent time series plots for mean capillary RBC SO_2_, hematocrit, velocity, and supply rate, respectively, for capillaries outside the micro-outlet edge at varying distances during low (7→2%) [O_2_] challenges. [O_2_] challenges consisted of 1 min at a baseline [O_2_] of 7% followed by 2 min at 2%. The average was taken from the entire first minute at 7% and the last 15 s at a 2% [O_2_]. **(A.2–A.4)** represents mean capillary RBC SO_2_ of capillaries within 100 µm from the micro-outlet edge (n = 41 capillaries), between 100–200 µm from the outlet edge (n = 34 capillaries), and greater than 200 µm from the outlet edge (n = 57 capillaries), respectively. **(B.2–B.4)** represents mean capillary hematocrit of capillaries within 100 µm from the micro-outlet edge (n = 30 capillaries), between 100–200 µm from the outlet edge (n = 38 capillaries), and greater than 200 µm from the outlet edge (n = 72 capillaries), respectively. **(C.2–C.4)** represents mean capillary RBC velocity of capillaries within 100 µm from the micro-outlet edge (n = 30 capillaries), between 100–200 µm from the outlet edge (n = 38 capillaries), and greater than 200 µm from the outlet edge (n = 72 capillaries), respectively. **(D.2–D.4)** represents mean capillary RBC supply rate of capillaries within 100 µm from the micro-outlet edge (n = 30 capillaries), between 100–200 µm from the outlet edge (n = 38 capillaries), and greater than 200 µm from the outlet edge (n = 72 capillaries), respectively. p values indicated in the figure with a *p* < 0.05 are considered significant. Box and whisker plots show minimum, median, maximum, and associated quartiles.

High [O_2_] challenges imposed on capillaries directly overlying 200 μm micro-outlets caused a small but significant increase in capillary hematocrit in vessels greater than 200 μm away from the outlet at 12% GEC [O_2_], 20.1% ± 9.6%, compared to baseline 7% GEC [O_2_], 19.1% ± 9.5% (*p* = 0.0172) (Figure 11). [O_2_] challenges imposed on capillaries directly overlying 200 μm micro-outlets caused significant changes in capillary hematocrit at 2% GEC [O_2_], 22.9% ± 10.2%, compared to baseline 7% GEC [O_2_], 20.7% ± 8.5% (*p* = 0.0022) (Figure 12). [O_2_] challenges imposed on capillaries directly overlying 200 μm micro-outlets caused a significant increase in capillary hematocrit in vessels less than 100 μm away from the outlet at 2% GEC [O_2_], 18.9% ± 10.7%, compared to baseline 7% GEC [O_2_], 17.8% ± 10.4% (*p* = 0.0348) (Figure 13).

[O_2_] challenges imposed on capillaries directly overlying 200 μm micro-outlets caused significant changes in capillary RBC velocity at 12% GEC [O_2_], 192.7 ± 107.5 µm/s, compared to baseline 7% GEC [O_2_], 207.5 ± 123.7 µm/s (*p* = 0.0370) (Figure 10). [O_2_] challenges imposed on capillaries directly overlying 200 μm micro-outlets caused significant changes in capillary RBC velocity at 2% GEC [O_2_], 293.1 ± 175.6 µm/s, compared to baseline 7% GEC [O_2_], 230.2 ± 141.0 µm/s (*p* < 0.0001) (Figure 12).

[O_2_] challenges imposed on 200 μm micro-outlets caused significant changes in capillary RBC velocity in vessels greater than 200 μm away from the outlet at 12% GEC [O_2_], 197.2 ± 117.8 µm/s compared to baseline 7% GEC [O_2_], 183.5 ± 116.5 µm/s (*p* = 0.0211) (Figure 11). [O_2_] challenges imposed on 200 μm micro-outlets caused a significant increase in capillary RBC velocity in vessels less than 100 μm away from the outlet at 2% GEC [O_2_], 207.8 ± 123.6 µm/s compared to baseline 7% GEC [O_2_], 182.0 ± 118.9 µm/s, in vessels between 100–200 μm away at 2% GEC [O_2_], 223.8 ± 122.3 µm/s, compared to baseline 7% GEC [O_2_], 189.1 ± 95.1 µm/s, and in vessels greater than 200 μm away from the outlet at 2% GEC [O_2_], 225.9 ± 143.8 µm/s compared to baseline 7% GEC [O_2_], 210.1 ± 119.8 µm/s (*p* < 0.0161) (Figure 13).

[O_2_] challenges imposed on capillaries directly overlying 200 μm micro-outlets caused a significant increase in capillary RBC supply rate at 2% GEC [O_2_], 17.3 ± 12.3 cells/s, compared to baseline 7% GEC [O_2_], 12.6 ± 10.2 cells/s (*p* < 0.0001) but not at 12% GEC [O_2_] (Figures 10, 12). [O_2_] challenges imposed on 200 μm micro-outlets caused significant changes in capillary RBC supply rate for vessels 200 μm away from the outlet edge at 12% GEC [O_2_], 9.6 ± 7.8 cells/s, compared to baseline 7% GEC [O_2_], 8.5 ± 6.9 cells/s (*p* = 0.0007) (Figure 11). [O_2_] challenges imposed on capillaries directly overlying 200 μm micro-outlets caused a significant increase in capillary RBC supply rate for vessels less than 100 μm away at 2% GEC [O_2_], 11.0 ± 9.3 cells/s, compared to baseline 7% GEC [O_2_], 9.0 ± 8.7 cells/s, in vessels in the 100–200 μm range from the outlet edge at 2% GEC [O_2_], 12.2 ± 8.7 cells/s, compared to baseline 7% GEC [O_2_], 9.1 ± 6.8 cells/s, and in vessels greater than 200 μm away at 2% GEC [O_2_], 11.2 ± 11.3 cells/s compared to baseline 7% GEC [O_2_], 9.5 ± 8.7 cells/s (*p* < 0.0248) (Figure 13).

**FIGURE 14:**
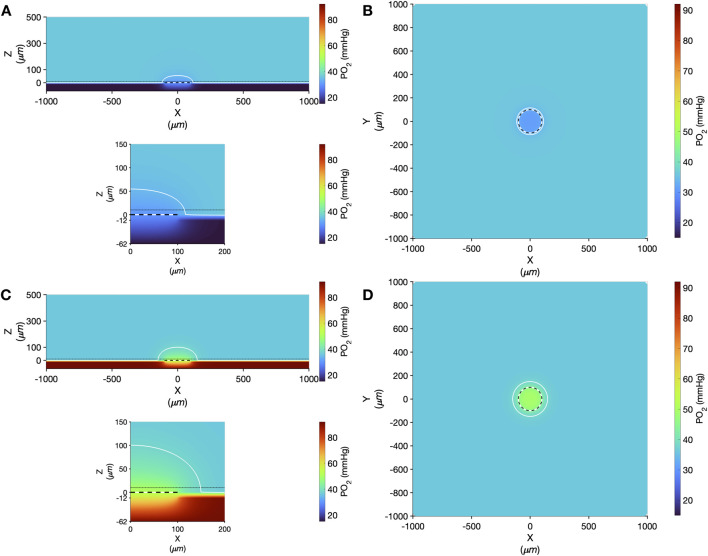
Oxygen transport simulation predicting tissue PO_2_ resulting from diffusional exchange between the tissue and gas exchange chamber via a 200 µm diameter thin-film micro-outlet device. In all panels the micro-outlet surface is indicated by the white and black dashed line. Simulation results for low [O_2_] challenges at 2% [O_2_] in the chamber are shown at the centre of the outlet in the XZ plane **(A)**. The dotted-dashed line in **(A)** indicates the location of the XY plane shown in panel B that is 10 µm into the tissue volume. Background tissue PO_2_ within the volume at a distance from the micro-outlet is 36.6 mmHg, with the iso-line in **(A–D)** delineating the volume of tissue overlying the micro-outlet that experiences a difference up to ±2 mmHg during the [O_2_] challenges. Simulation results for high [O_2_] challenges at 12% [O_2_] in the chamber are shown at the middle of the outlet in the XZ plane **(C)** and at a depth of 10 µm into the tissue volume in the XY plane **(D)**. The lower panel in A and C show a magnified view of the interface between the micro-outlet device and the overlying tissue.

Computer simulation results showing the steady state PO_2_ within the tissue and 200 μm micro-outlet device for high (12%) and low (2%) [O_2_] challenges are presented in Figure 14. Simulations of steady state conditions while the gas exchange chamber [O_2_] is held at 2% predicted that PO_2_ within the tissue volume would be decreased by >2mmHg up to a radial distance of 16.5 μm beyond the micro-outlet edge, and up to 54.5 μm vertically into the tissue volume in the Z-axis along the centre of the circular micro-outlet (Figure 14). Simulations for high (12%) [O_2_] within the gas exchange chamber predicted an increase in tissue PO_2_ of >2 mmHg up to a radial distance of 49.5 μm beyond the micro-outlet edge, and a depth into the tissue volume up to 101.5 μm (Figure 14).

### 3.6 600 and 1,000 µm micro-outlet data

[O_2_] oscillations and challenges were also imposed using 600 and 1,000 µm micro-outlet devices to study how larger exchange surfaces may impact the resulting microhemodynamic responses. The data from these larger micro-outlet devices is largely comparable to the findings presented above for 200 and 400 µm micro-outlets. To provide additional context we briefly discuss the responses to low (2%) [O_2_] challenges in the 600 and 1,000 µm micro-outlets devices below.

Low [O_2_] challenges imposed using 600 μm micro-outlets caused a significant decrease in capillary RBC SO_2_ at 2% GEC [O_2_], 50.2% ± 18.33%, compared to baseline 7% GEC [O_2_], 72.4% ± 11.3% (*p* < 0.0001) (Supplementary Figure S3). Low [O_2_] challenges caused significant increases in capillary hematocrit at 2% GEC [O_2_], 25.54% ± 10.82%, compared to baseline 7% GEC [O_2_], 21.36% ± 10.93% (*p* < 0.0001); capillary RBC velocity at 2% GEC [O_2_], 291.1 ± 182.7 μm/s, compared to baseline 7% GEC [O_2_], 218.1 ± 149.4 μm/s (*p* < 0.0001); and supply rate at 2% GEC [O_2_], 18.4 ± 14.8 cells/s, compared to baseline 7% GEC [O_2_], 12.3 ± 11.9 cells/s (*p* < 0.0001) (Supplementary Figure S3).

Computer simulation results showing the steady state PO_2_ within the tissue and 600 μm micro-outlet device for high (12%) and low (2%) [O_2_] challenges are presented in Supplementary Figure S4. Simulations of steady state conditions while the gas exchange chamber [O_2_] is held at 2% predicted that PO_2_ within the tissue volume would be decreased by >2 mmHg up to a radial distance of 16.5 μm beyond the micro-outlet edge, and up to 61.4 μm vertically into the tissue volume in the Z-axis along the center of the circular micro-outlet (Supplementary Figure S4). Simulations for high (12%) [O_2_] within the gas exchange chamber predicted an increase in tissue PO_2_ of >2 mmHg up to a radial distance of 64.5 μm beyond the micro-outlet edge, and a depth into the tissue volume up to 131.7 μm (Supplementary Figure S4).

Low [O_2_] challenges imposed using 1,000 μm micro-outlets caused significant decrease in capillary RBC SO_2_ at 2% GEC [O_2_], 45.73% ± 19.75%, compared to baseline 7% GEC [O_2_], 71.15% ± 15.28% (p < 0.0001) (Supplementary Figure S5). Low [O_2_] challenges caused significant increases in capillary hematocrit at 2% GEC [O_2_], 25.81% ± 9.12%, compared to baseline 7% GEC [O_2_], 19.34% ± 10.04% (*p* < 0.0001); capillary RBC velocity at 2% GEC [O_2_], 261.2 ± 177.0 μm/s, compared to baseline 7% GEC [O_2_], 166.6 ± 123.7 μm/s (*p* < 0.0001); and RBC supply rate at 2% GEC [O_2_], 16.80 ± 12.70 cells/s, compared to baseline 7% GEC [O_2_], 8.23 ± 7.40 cells/s (*p* < 0.0001) (Supplementary Figure S5).

Computer simulation results showing the steady state PO_2_ within the tissue and 1000 μm micro-outlet device for high (12%) and low (2%) [O_2_] challenges are presented in Supplementary Figure S6. Simulations of steady state conditions while the gas exchange chamber [O_2_] is held at 2% predicted that PO_2_ within the tissue volume would be decreased by >2 mmHg up to a radial distance of 23.3 μm beyond the micro-outlet edge, and up to 67.9 μm vertically into the tissue volume in the Z-axis along the center of the circular micro-outlet (Supplementary Figure S6). Simulations for high (12%) [O_2_] within the gas exchange chamber predicted an increase in tissue PO_2_ of >2 mmHg up to a radial distance of 59.3 μm beyond the micro-outlet edge, and a depth into the tissue volume up to 120.4 μm (Supplementary Figure S6).

## 4 Discussion

In this study, we developed and validated a novel thin-film micro-outlet device to deliver highly localized O_2_ perturbations to regions of skeletal muscle tissue with simultaneous quantification of blood flow responses using intravital video microscopy. The knowledge obtained from previous designs, *in vivo* experiments, and the size of a single microvascular unit in rodents (∼150 × 200 μm), informed our device design process (Ghonaim et al., 2011; 2013; 2021; Sové et al., 2021; Ellis et al., 2012). Laser-cut PVDC gas-impermeable film was used to pattern thin-film micro-outlets within our device to deliver oxygen perturbations into microscale tissue regions. The device was coupled with computer-controlled mass flow meters connected to a GEC for imposing [O_2_] perturbations to the surface of the EDL muscle overlying an inverted microscope setup. To validate the ability of our device to manipulate tissue [O_2_] via the GEC to the muscle, RBC SO_2_ was analyzed in capillaries overlying the outlet and at various distances away from the outlet edge using IVVM and offline MATLAB software (Ellis et al., 1990; 1992; 2012; Japee et al., 2004; Japee et al., 2005; Fraser et al., 2012; Ghonaim et al., 2021).

Other groups have previously achieved the spatial specificity required to alter blood flow through induced microvascular RBC SO_2_ changes *in vivo* (Frisbee and Lombard, 2002; Frisbee et al., 2002; Ghonaim et al., 2011; 2021; Sové et al., 2021). Superfusion solutions of varying O_2_ levels have been used to stimulate arterioles to either vasoconstrict or vasodilate, depending on the [O_2_] bathing the tissue surface (Duling and Berne, 1970a; Frisbee and Lombard, 2002; Frisbee et al., 2002; Riemann et al., 2011; Charter et al., 2018). Compared to a micro-outlet device coupled to a gas exchange chamber that directly stimulates individual microvascular networks of an isolated muscle, this stimulated area in a bathed muscle preparation is much larger, affecting approximately 40-fold the microvascular surface area (Ghonaim et al., 2011; Frisbee and Lombard, 2002; Duling and Berne, 1970a). When the whole muscle is targeted in this type of preparation, multiple levels of the vasculature and associated regulatory mechanisms may be affected (Duling and Berne, 1970a; Frisbee and Lombard, 2002; Charter et al., 2018; Russell McEvoy et al., 2022). Therefore, micro-outlet devices offer more spatially constrained tissue manipulations at precise concentrations that can be changed dynamically or maintained for extended durations.

Unlike isolated muscle preparations, superfusion setups do not completely isolate the muscle from the environment (Tyml and Budreau, 1991; Riemann et al., 2011). Instead, a fixed gas concentration is maintained within the solution that bathes it (Riemann et al., 2011; Charter et al., 2018). These solutions are typically equilibrated with 0% [O_2_] which may interfere with the interpretation of muscle blood flow, as regardless of other stimuli, the regulatory system will adjust to facilitate matching O_2_ supply and demand (Kindig et al., 2002). Due to the superfusate being continuously washed away and replenished, other regulatory molecules, such as nitric oxide, may also be depleted, affecting physiological pathways and basal tone (Jackson, 2016). This may lead to confounding results in O_2_ reactivity studies (Jackson, 2016). In general, oxygen has a low solubility in water, thus superfusion solutions have a finite carrying capacity which may serve as a further limiting factor. Using gas exchange chambers to stimulate the overlying muscle mitigates the consideration of O_2_ solubility properties as manipulations are made in the gas phase and delivered over short diffusion distances across a gas permeable membrane (Ellis et al., 2012; Ghonaim et al., 2011; 2021; Sové et al., 2021; Russell McEvoy et al., 2022). These limitations further support the use of gas-based microfluidic approaches for studying microvascular oxygen regulation.

Through studies focused on O_2_ reactivity at the microvascular level, it has been shown that when enough tissue surface is stimulated, an appropriate blood flow response will occur; a further insight on the location for O_2_ sensing (Ellis et al., 2012; Ghonaim et al., 2011; 2013; 2021; Sové et al., 2021; Russell McEvoy et al., 2022). In this study, we aimed to quantify blood flow responses in individual capillary networks to gain novel insights into O_2_ sensing at the capillary level. Previously, a 100 μm diameter circular outlet was shown to change RBC SO_2_ in 1-2 overlying capillaries but did not stimulate a blood flow response (Ghonaim et al., 2011). RBC SO_2_ manipulations in capillary networks overlying 1,000 by 200 μm micro-outlet elicited a blood flow response, suggesting that O_2_ sensing and control is, at least in part, localized to capillaries (Ghonaim et al., 2021). Subsequently a similar microvascular preparation with a 400 by 200 μm micro-outlet was shown to elicit a substantial RBC SO_2_ change in capillaries overlying their micro-outlets and to elicit a blood flow response by activating O_2_-mediated responses (Sové et al., 2021). Taken together, these studies demonstrate the efficacy of micro-outlet devices to manipulate the tissue microenvironment, and establish that such [O_2_] perturbations directed at capillaries are capable of provoking blood flow responses, yet such responses may be dependent on the number of stimulated vessels or a critical scale of tissue volume (Ghonaim et al., 2011; 2021; Sové et al., 2021).

Radial diffusion is an important characteristic of gas-based micro-outlet devices as it relates to their achievable spatial specificity. Importantly, the distance a stimulus extends into the tissue will affect how well constrained imposed [O_2_] perturbations are. Mathematical modelling and empirical studies have been used to describe the distribution of PO_2_ in tissue overlying the micro-outlets (Ghonaim et al., 2013). The results from Ghonaim et al. suggested that imposed PO_2_ changes from the GEC diffused to capillaries overlying the outlet and to capillaries up to 100 μm away. Still, logically this change in PO_2_ declined over distance as the tissue returned to the mean background tissue PO_2_ (Ghonaim et al., 2013). Unfortunately, this mathematical model did not consider the gas-permeable PDMS layer directly interfaced with the muscle of interest (Ghonaim et al., 2013; Sové et al., 2021). Therefore, O_2_ perturbations in previous devices diffused more readily through the PDMS layer (due to the higher diffusivity and solubility of oxygen in PDMS), and into the muscle tissue, causing the perturbation to spread capillaries at significant distances away from the outlet (Sové et al., 2021). Therefore, this led to an underestimated radial diffusion, PO_2_ distribution, and spatial specificity of the micro-outlets in the tissue (Ghonaim et al., 2011; 2013). Further *in vivo* studies were performed, and a new model that incorporated the PDMS layer was created to aid in interpreting the results from Sové et al.’s 400 by 200 μm rectangular micro-outlet device (Sové et al., 2021). This new model and its associated *in vivo* RBC SO_2_ data from capillaries at various distances away from the micro-outlets provided novel insights on the extent of O_2_ spread within the PDMS layer in their device (Sové et al., 2021). The spreading effect increased this previous outlets’ overall area impacted to greater than 614 by 434 μm, meaning tissue and capillaries farther than 200 μm from the outlet edge experienced significant O_2_ changes (Sové et al., 2021). A comparison of low [O_2_] simulation results for the micro-outlet device geometry used in the present study and that used in Sové et al., 2021 is provided in Supplementary Figure S7 for reference. Therefore, the O_2_ perturbations permeate through a larger volume of tissue and vasculature than originally proposed, potentially integrating direct interactions with regulatory mechanisms in higher order vessels such as arterioles.

To determine if similar control responses would occur in a device that more precisely stimulated regions of muscle tissue, device modifications and further experiments were deemed necessary (Sové et al., 2021). With these insights in mind, our design for circular micro-outlets with diameters of 200 and 400 μm were fabricated to better determine the minimum scale of O_2_ sensing and control. One of the critical device modifications that was made in the present study was changing the position of the gas permeable layer to be in contact with the GEC, and only interfaced with the muscle at the surface of the patterned micro-outlets (Ghonaim et al., 2011; 2013; 2021; Sové et al., 2021). The purpose of this design modification was to eliminate or mitigate the effects of O_2_ spreading within the PDMS that exacerbates remote changes to tissue [O_2_] that confounded previous studies (Sové et al., 2021).

In addition to providing greater spatial specificity, our thin-film micro-outlet devices provide superior optical properties compared to previous devices (Sové et al., 2021), which allowed us to observe and analyze capillary responses inside and outside the outlets simultaneously (Figure 2). Our micro-outlet device was designed with a thin PVDC film for the gas impermeable layer, whereas previous groups used thin glass substrates of different thicknesses ranging from 80–180 μm (Ghonaim et al., 2011; 2013; 2021; Sové et al., 2021). When using glass substrates with air filled micro-outlets, the disparity in refractive index causes capillaries overlying the outlets to resolve at a different focal plane than those outside, even though they are at the same tissue depth (Sové et al., 2021; Figure 2). This is also a disadvantage for quantifying capillary responses overlying and outside the outlets, as the recordings must be captured separately, resulting in temporally unpaired measurements. The PVDC film employed in our device produces a much thinner exchange membrane, provides excellent optical clarity, and as a result, the ability to analyze responses of capillaries overlying and outside the outlets simultaneously during [O_2_] oscillations and challenges.

### 4.1 Oxygen oscillations

In our study, we altered muscle [O_2_] concentrations using a gas-based micro-outlet device and GEC that was directly interfaced with the EDL muscle. As expected, the 4-min [O_2_] oscillations in the GEC were able to induce rapid and profound alterations in capillary RBC SO_2_ in capillaries overlying the 400 μm micro-outlets without affecting the SO_2_ in capillaries outside the outlets. The SO_2_ measurements validated our device’s ability to impose rapid localized gas-based perturbations without altering the tissue microenvironment in neighboring vessels outside the micro-outlet. Capillary RBC SO_2_ changes in the tissue occurred rapidly, reaching their mean peak responses within 12 s at 12% [O_2_] (86.9%) and 11 s at 2% [O_2_] (43.5%) (Figure 3).

Significant hemodynamic responses in capillaries directly overlying the 400 μm micro-outlets occurred without affecting neighboring vessels adjacent to the micro-outlet during [O_2_] oscillations (Figures 3, 4). Specifically, significant differences were observed for RBC supply rate, velocity, and hematocrit levels between 12% and 2% [O_2_] oscillations (Figure 3). Interestingly, there was a significant difference in mean RBC supply rate between the first, baseline 7% [O_2_] and the last 7%, where we expected to see similar flow states as both time periods were collected at the same baseline [O_2_]. This data suggests that 1 min at baseline 7% may not be enough time for the flow to normalize after it undergoes high and low 1-min [O_2_] oscillations; this is interpreted further below.

During [O_2_] oscillations, hematocrit levels were only significantly different between 12% and 2% GEC [O_2_] (Figure 3), suggesting hematocrit levels do not modulate readily to [O_2_] perturbations slightly above or below baseline conditions. It was presented in the literature by Kindig et al. and supported by Russell McEvoy et al. that transient changes in hematocrit are a result of diameter changes in higher order arterioles altering hematocrit levels of downstream vessels based on the Fåhræus effect (Fåhræus and Lindqvist, 1931; Barbee and Cokelet, 1971; Pries et al., 1986; Kindig et al., 2002; Russell McEvoy et al., 2022). Since the microvascular region affected is highly confined in our 200 and 400 μm micro-outlets, it is unlikely that higher order arterioles are stimulated; thus, vasoactive responses in terminal arterioles would not result in significant changes in hematocrit during [O_2_] oscillations. Therefore, the number of capillaries stimulated by O_2_ perturbations was enough to elicit a flow increase in response to low [O_2_] conditions but not a consistent decrease at high [O_2_] conditions.

There were significant increases in velocity and supply rate between the initial 1 min 7% [O_2_] and the second 7% [O_2_] condition, which suggests flow did not return to baseline after increasing during the 2% [O_2_]. A study published by our group using full tissue surface perturbations described time transients during 2*→*7% [O_2_] challenges with their associated mean peak responses in capillary RBC velocity (t = 77 s), hematocrit, (t = 105 s), and supply rate (t = 76 s) (Russell McEvoy et al., 2022). Such results suggest that after a 1-min oscillation at 2%, the baseline hemodynamic responses to 7% [O_2_] did not have enough time to provoke a full response in the 1-min oscillation. Therefore, this may explain why we found a significant increase between our first and last baseline 7% minute.

Capillaries analyzed in our 400 μm diameter micro-outlets did not experience a significant decrease in hemodynamic values between baseline 7% and 12% [O_2_] during oscillations. There are a few potential reasons for this result. Compared to previous studies, our results suggest the chosen outlet sizes could not perturb the vasculature sufficiently to initiate O_2_ sensing mechanisms to react when [O_2_] was increased to 12% (Ghonaim et al., 2011; 2013; Sové et al., 2021; Russell McEvoy et al., 2022). Russell McEvoy et al., provided evidence to show that 7*→*12% and 7*→*2% [O_2_] changes provoke regulatory responses that are asymmetric and that when [O_2_] is increased from baseline, the hemodynamic regulatory response is not as profound as it is when [O_2_] is decreased to 2%. This is possibly due to a more robust change in capillary RBC SO_2_ between 7*→*2% GEC [O_2_] which provoked a 29.5% decrease in RBC SO_2_ compared to a 19.7% increase in RBC SO_2_ for the 7*→*12% GEC [O_2_] (Russell McEvoy et al., 2022). Thus, the more profound the RBC SO_2_ change, the greater the elicited flow response. Our oscillation data supports this conclusion as we observed significant hemodynamic increases during low 2% [O_2_] oscillations compared to baseline 7%. Furthermore, in some previous micro-outlet studies, the baseline GEC [O_2_] used was 5% (Ghonaim et al., 2011; 2021; Sové et al., 2021), and high [O_2_] was 12% or 20%, creating a considerably more forceful step change in [O_2_] compared to the present study (Ghonaim et al., 2011; 2021; Sové et al., 2021). Significant hemodynamic differences were found during similar high [O_2_] conditions in previous studies, but their oscillations were longer than in our current work, with high and low step changes occurring for 2 min each (Sové et al., 2021; Ghonaim et al., 2021). Therefore, inducing sequential high and low [O_2_] perturbations may interfere with the ability of the control system to reach a peak hemodynamic response over the observation period. It is also plausible that the signaling pathways linking RBC SO_2_ with functional hemodynamic responses are non-linear, which could be a result of enhanced ATP release at lower SO_2_, luminal ATP concentration dependence to trigger endothelial cell hyperpolarization, or other as yet undefined oxygen-dependent mechanisms within the transduction pathway. Lastly, the location of terminal arterioles supplying the network of interest and their distance from an outlet may influence the ability to modulate blood flow. Studies that stimulated larger areas of muscle tissue are more likely to interact with terminal arterioles, or higher vasculature (Ghonaim et al., 2021; Sové et al., 2021; Russell McEvoy et al., 2022). Modulating tissue [O_2_] both at a terminal arteriole and its downstream capillary network may result in a more robust or rapid regulatory response.

### 4.2 Oxygen challenges

As expected, high (7*→*12%) and low (7*→*2%) [O_2_] challenges from the gas exchange chamber caused rapid and profound alterations in capillary RBC SO_2_ in capillaries overlying the 400 μm micro-outlets without causing significant changes in capillary RBC SO_2_ outside the outlets (Figures 5–8). Additionally, capillaries less than 100 μm away from the 200 μm outlets experienced significant but comparatively small RBC SO_2_ changes during high [O_2_] challenges (Figure 11). Compared to the RBC SO_2_ change experienced by capillaries directly overlying the micro-outlet, capillaries less than 100 μm away from the outlet edge changed from 42.2% to 47.2%, whereas the capillaries overlying the micro-outlet experienced an increase from 60.7% to 80.0% RBC SO_2_ (Figures 10, 11). Our data shows that the area of effect for our 200 μm diameter micro-outlets is within 100 μm of the micro-outlet edge when imposing 2-min [O_2_] challenges. Interestingly, for the 2% [O_2_] condition, our model predicted changes in tissue PO_2_ of >2 mmHg up to a radial distance of 17 μm from the 400 μm micro-outlet, though we did not find any significant changes in capillary RBC SO_2_ in the <100 μm distance range. This is likely due to the small number of capillaries available to be analyzed in the region within 17 μm of the outlet edge, with a larger proportion of measurements in the <100 μm distance bin coming from vessels 17–100 μm from the micro-outlet. Furthermore, since the perturbation in tissue PO_2_ drops exponentially with distance from the micro-outlet, capillaries at a distance are more resistant to changes in SO_2_ as they are supplied with ‘fresh’ RBCs from tissue volumes that are unperturbed by the micro-outlet [O_2_].

The 7*→*2% [O_2_] challenges induced significant hemodynamic responses in capillaries directly overlying 400 μm micro-outlets, as determined by increased mean RBC supply rate and velocity (Figure 7). Significant hemodynamic decreases were observed during high [O_2_] challenges in capillaries overlying the 400 μm diameter outlets (Figure 5). This is an exciting finding as we did not observe significant responses between 7% and 12% during our 4-min oscillations. This finding supports our interpretation of a time-dependent effect related to stimulating O_2_ regulatory mechanisms in high [O_2_] areas in small muscle tissue regions. Significant hematocrit changes occurred during low [O_2_] challenges but not during high [O_2_] challenges.

Although some flow responses did occur in vessels outside the 200 μm outlets, there were no corresponding mean RBC SO_2_ changes in these vessels. The changes in flow outside the 200 μm diameter micro-outlets may be a result of conducted signaling from vasculature overlying the micro-outlets, directly connected with vessels at a distance (Collins et al., 1998; McCullough et al., 1997; Bagher and Segal, 2011). If stimulating a capillary network overlying the micro-outlet affected capillary networks connected to the same feed arteriole situated outside the outlet, it would support the role of conducted signaling in the response (Collins et al., 1998; Ellsworth, 2004; Ellsworth et al., 2009; Ellis et al., 2012; Ghonaim et al., 2011). In comparison, Sové et al., did not obtain robust hemodynamic changes in capillaries at a distance from their outlets, though details regarding this data were not reported. Still, they provide data showing profound changes in RBC SO_2_ in capillaries measured at three distances reaching farther than 200 μm from the outlet edge. Based on the significant SO_2_ changes found, it is surprising that they did not observe corresponding flow changes in these capillaries as they did in capillaries overlying outlets. However, based on our knowledge of the study, this was due to a relatively small number of remote capillaries available to be sampled for comparisons (Sové et al., 2021).

Hemodynamic responses were observed in capillaries outside the 400 μm diameter outlets at distances farther than 100 μm away but not in vessels less than 100 μm away during low [O_2_] challenges. This observation showed a 25% supply rate increase in capillaries 200 μm away from the outlet compared to a 65% mean supply rate increase in vessels directly crossing the outlets (Figures 7, 8). These hemodynamic changes remote to the micro-outlets may be due to conducted signaling between interconnected vasculature, as mentioned above. In our devices and in previous devices, multiple micro-outlets are in contact with the muscle within a relatively small region of the tissue surface (Sové et al., 2021). In our devices, adjacent micro-outlets are separated by 1,000 μm. Since the microcirculation is highly interconnected, vessels overlying the micro-outlets that experience a supply rate change may also induce a supply rate change in vessels at a distance from the outlet; one that may be unrelated to the local tissue micro-environment in these remote vessels. Multiple micro-outlets may stimulate responses remote to the confined stimulated region, which is more likely to cause significant supply rate changes in outside vessels.

It has been proposed repeatedly in the literature that capillaries are involved in oxygen-mediated blood flow regulation (Ellsworth et al., 1995; Ellsworth et al., 2009; Ellis et al., 2012; Dietrich and Tyml, 1992; Song and Tyml, 1993; Ghonaim et al., 2011; Ghonaim et al., 2013; Ghonaim et al., 2021; Sové et al., 2021; Lamb et al., 2021). Capillaries are suggested to play a role in sensing local O_2_ conditions via SO_2_-dependent ATP release from erythrocytes which then initiates conducted signaling to arterioles upstream (Collins et al., 1998; Dietrich et al., 2009; Arciero et al., 2008; Ellsworth et al., 2009; Dora, 2017). Our observations further demonstrate that the magnitude of capillary RBC supply rate responses are closely coupled with capillary RBC SO_2_. In the case of our micro-outlet devices, the fixed PO_2_ condition at the exchange surface effectively clamps capillary RBC SO_2_, which, in conjunction with the resulting supply rate response, imposes a static stimulus to the regulatory system that is analogous to a highly localized change in tissue O_2_ consumption. *In situ*, increases in whole muscle O_2_ consumption during exercise are similarly accommodated by proportional increases in blood flow and O_2_ extraction, thus matching total tissue O_2_ demand with supply (Andersen and Saltin, 1985; Casaburi et al., 1989). The present study provides direct evidence that O_2_ demand-supply matching occurs at or below the scale of single capillary units, and implies that fine regulatory control of blood flow distribution within tissues is necessary to satisfy changes in overall O_2_ demand.

**FIGURE 15:**
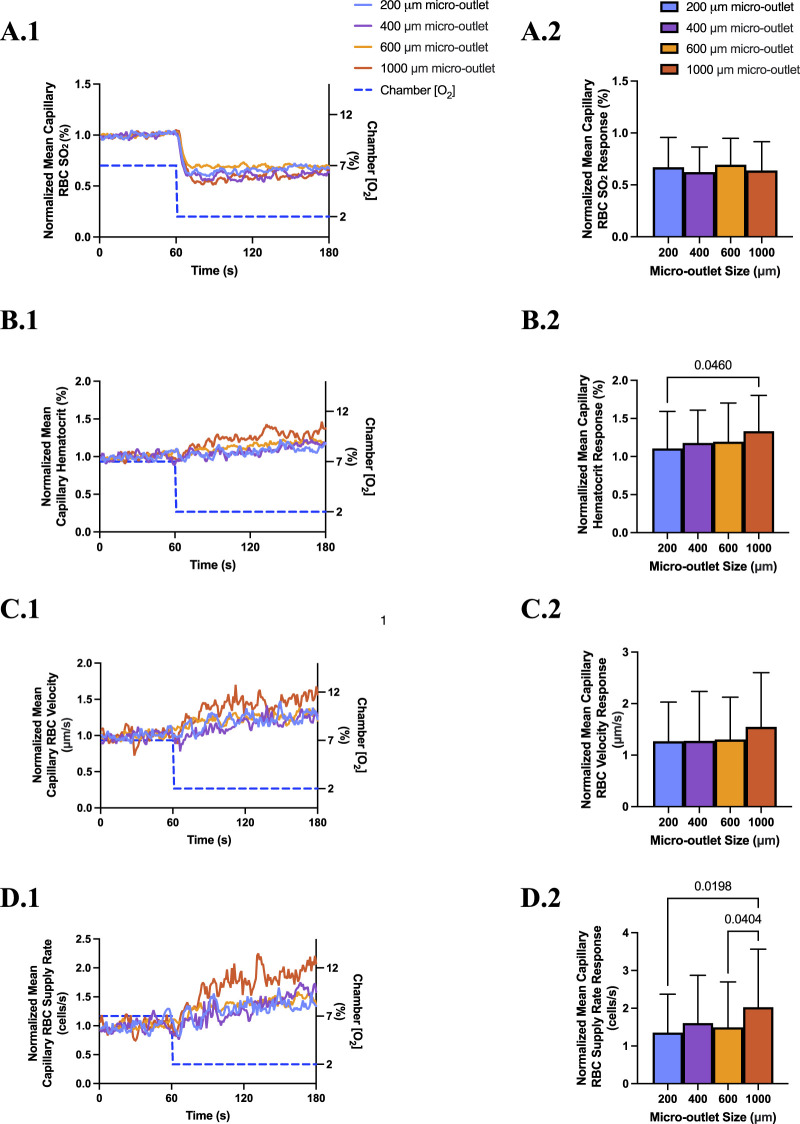
Normalized mean capillary red blood cell (RBC) oxygen saturation (SO_2_) and hemodynamic responses for capillaries directly overlying the 200, 400, 600, and 1,000 µm micro-outlets in response to low (7*→* 2%) oxygen concentration ([O_2_]) challenges. Each channel was normalized to the mean of the last 10 s of the baseline period. [O_2_] challenges began with 1-min baseline [O_2_] at 7% followed by 2 min at 2%. Time series plots are displayed in **(A.1–D.1)** comparing the normalized mean capillary RBC SO_2_ **(A.1)** (n = 43, 200 μm; n = 36, 400 μm; n = 79, 600 μm; n = 61, 1,000 µm), hematocrit **(B.1)**, velocity **(C.1)**, and supply rate **(D.1)**, for capillaries overlying each of the four micro-outlet sizes during low [O_2_] challenges (for all hemodynamic channels n = 53, 200 μm; n = 36, 400 μm; n = 93, 600 μm; n = 73, 1,000 µm). **(A.2–D.2)** represent the normalized mean responses for the last 15 s at 2% [O_2_] compared to the normalized baseline (7%) for capillary RBC SO_2_, hematocrit, velocity, and supply rate, in capillaries directly overlying the four micro-outlet sizes. Results show that blood flow responses are larger in magnitude for capillaries overlying larger diameter outlets versus smaller outlets. *p* values indicated in the figure with a *p* < 0.05 are considered significant, error bars show standard deviation of the mean.

It has also been hypothesized that the magnitude of the flow responses observed in relation to [O_2_] perturbations is proportional to the number of capillaries and endothelial cells stimulated within a network (Ellis et al., 2012; Ghonaim et al., 2013; 2021; Sové et al., 2021). Our results for different outlet sizes support this hypothesis, as in 7*→*2% [O_2_] challenges we observed a 38% increase in capillary RBC supply rate in the 200 μm diameter outlets, a 65% increase in 400 μm outlets, and a 104% increase in 1,000 μm, despite similar changes in capillary RBC SO_2_ (Figure 15). Indeed, the normalized comparison of low O_2_ challenge responses for the four sizes of micro-outlets suggests that larger micro-outlets provoke a greater and more vigorous responses (Figure 15). During high [O_2_] challenges, our 200 μm diameter micro-outlets did not provoke a significant decrease in capillary RBC supply rate, but mean capillary RBC supply rate did have a significant decrease of 12% in the 400μm micro-outlets. Endothelial cells in skeletal muscle are approximately 104 μm long, and microvascular units are typically 150 × 200 μm in rodents (Adamson, 1993; Yuan and Rigor, 2010), so a larger outlet would be expected to stimulate more endothelial cells to hyperpolarize and contribute to a conducted response from [O_2_] changes. Therefore, the larger the micro-outlet device and muscle tissue area stimulated by [O_2_] perturbations, the greater the potential flow response. This is likely due to the integration of conducted signals reaching arterioles reflecting hyperpolarization of the capillary endothelium from multiple capillaries that the arteriole supplies (McCullough et al., 1997; Collins et al., 1998; Lidington et al., 2002; Dietrich et al., 2009; Ellis et al., 2012; Dora, 2016). Therefore, the magnitude of the flow response should be at least in part reflected by the number of endothelial cells and individual capillaries influenced by the micro-outlet perturbation (Ghonaim et al., 2011; 2013), and not just the change in capillary RBC SO_2_. Furthermore, in 100 μm outlet affecting 1-2 capillaries via [O_2_] perturbations did not elicit a flow response (Ghonaim et al., 2011), but our 200 μm micro-outlet affecting approximately double the capillaries and endothelial cells, did yield significant hemodynamic responses. Therefore, our results support the concept of spatially summative responses and provide evidence for capillary level O_2_ sensing.

Our significant capillary hemodynamic responses from 200 μm diameter micro-outlets represent a promising means to manipulate blood flow via [O_2_] at the capillary level without directly stimulating higher levels of the vasculature. As discussed above, flow responses observed in our small, circular 200 μm diameter micro-outlets indicate that individual capillary networks can sense O_2_ and precisely modulate local blood flow in response. In previous studies, micro-outlet devices could not achieve a blood flow response to a stimulus of this small size, therefore, our findings provide novel insights into the scale of O_2_ sensing. Future studies should aim to quantify the O_2_ flux from capillaries overlying the micro-outlet device to more directly describe how changes in O_2_ extraction in the stimulated capillaries relate to the modulation of supply rate, and hence O_2_ supply at this scale.

In conclusion, we have developed and validated a thin-film micro-outlet device that can elicit [O_2_] perturbations within highly confined regions of skeletal muscle tissue, effectively modulating local capillary RBC SO_2_ and provoking corresponding flow responses in micro-outlets as small as 200 μm in diameter. Our novel micro-outlet devices have also demonstrated superior optical properties that allow for temporally matched comparisons between capillaries directly overlying the micro-outlet and vessels at a distance from the stimulus.

In future studies, our device can be used to obtain the high level of spatial specificity necessary to interrogate capillary level regulation mechanisms using multi-modal approaches, such as intravascular pharmacological interventions. Our modular device can easily be modified to address a wide range of research questions and is suitable for future studies to target various levels of microcirculation and specific mechanisms responsible for oxygen-mediated blood flow regulation.

## Data Availability

The raw data supporting the conclusions of this article will be made available by the authors, without undue reservation.
