# Supplementary material for: Development of thin-film micro-outlets for spatially constraining local PO2 perturbations to capillaries in vivo
Source: Front Physiol. 2025 Jul 9;16:1575776. doi: 10.3389/fphys.2025.1575776 (PMC12283586; doi:10.3389/fphys.2025.1575776)
Supplement: Supplementary file 1 [file DataSheet1.pdf]

Supplementary Material

Table S1

Parameters used in mathematical model

Parameter	Value	References
D_{tissue}	2.41 x 10^{-5} cm^2/s	Bently et al., 1993
K_{tissue}	3.89 x 10^{-5} mL O_2/mL/mmHg	Mahler et al., 1985
D_{PDMS}	3.40 x 10^{-5} cm^2/s	Merkel et al., 2000
k_{PDMS}	1.32 x 10^{-5} mL O_2/mL/mmHg	Shiku et al., 2006
Dk_{PVDC}	1.04 x 10^{-13} (cm^2/s)*(mL O_2/mL/mmHg)	Asahi Kasei Corporation, 2024
M_0	1.57 x 10^{-4} mL O_2/mL/s	Sullivan and Pittman, 1984
q	30 mmHg/s	Goldman, 2008
p_b	42 mmHg	Goldman, 2008
p_{50}	0.5 mmHg	Honig and Gayeski, 1982

Supplemental Video S2

Intravital video of microvascular blood flow showing the change in capillary red blood cell (RBC) oxygen saturation (SO_2) during a low oxygen concentration ([O_2]) challenge, and the resulting hemodynamic response in terms of capillary RBC supply rate in capillaries directly overlying the micro-outlet exchange surface. [O_2] in the microfluidic gas exchange chamber is held constant at 7% [O_2] for the first minute before being abruptly changed to 2% for the remaining two minutes. The dashed circle indicates the location of the 400 µm diameter micro-outlet in the intravital video.

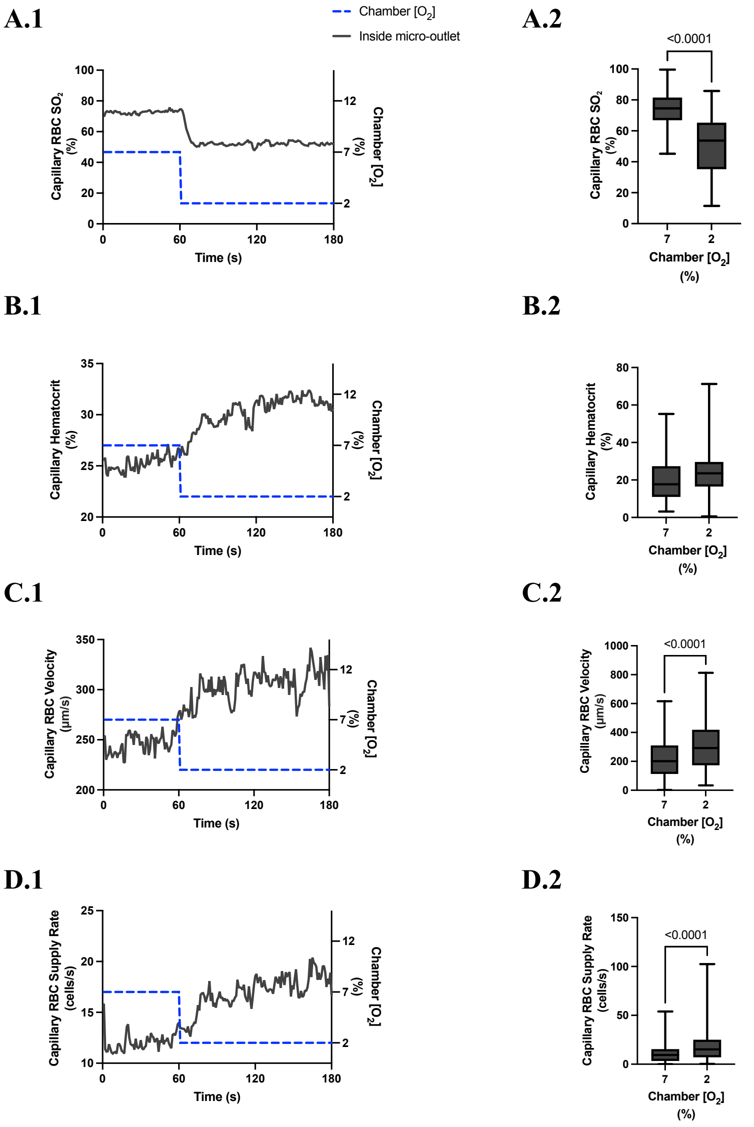

Figure S3

Capillary red blood cell (RBC) oxygen saturation (SO_2) and hemodynamic responses in capillaries directly overlying the 600 µm micro-outlet in response to low oxygen concentration ([O_2]) challenges. [O_2] challenges began with one-minute baseline [O_2] at 7% followed by two minutes at 2%. Time series plots are displayed in panel A.1 – D.1 for mean capillary RBC SO_2 (A.1), hematocrit (B.1), velocity (C.1), and supply rate (D.1), for capillaries overlying the micro-outlet during low [O_2] challenges. For panel A.2 – D.2, the average was taken from the entire first minute at 7% and the last 15 seconds at 2% [O_2]. Panel A.2 – D.2 represent mean RBC SO_2 (n = 79 capillaries), hematocrit, velocity, and supply rate (n = 93 capillaries), respectively, for capillaries directly overlying the 200 µm micro-outlet. p values indicated in the figure with a $p < 0.05$ are considered significant. Box and whisker plots show minimum, median, maximum, and associated quartiles.

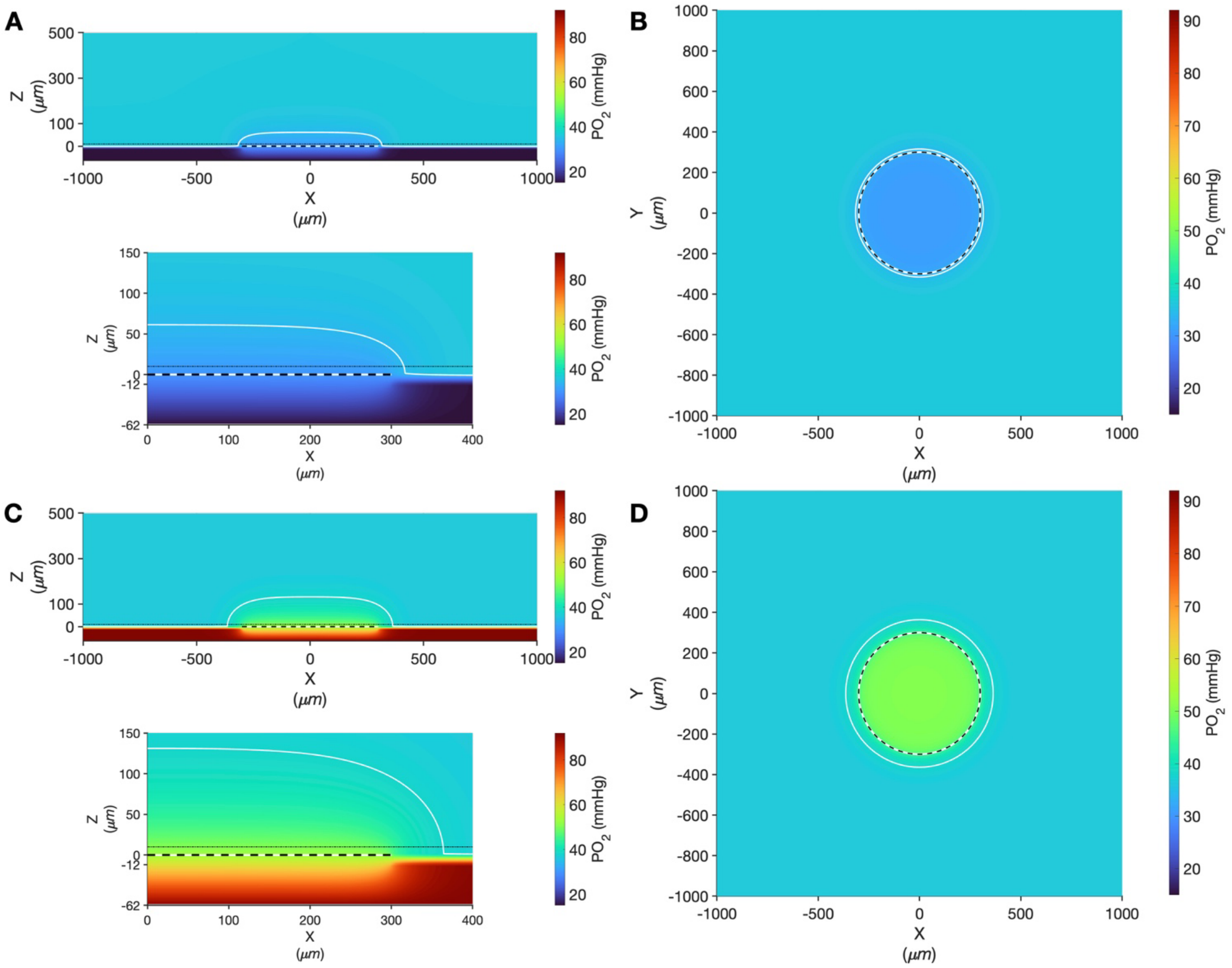

Figure S4
Oxygen transport simulation predicting tissue PO_2 resulting from diffusional exchange between the tissue and gas exchange chamber via a 600 µm diameter thin-film micro-outlet device. In all panels the micro-outlet surface is indicated by the white and black dashed line. Simulation results for low oxygen concentration ($[O_2]$) challenges at 2% $[O_2]$ in the chamber are shown at the center of the outlet in the XZ plane (A). The dotted-dashed line in A indicates the location of the XY plane shown in panel B that is 10 µm into the tissue volume. Background tissue PO_2 within the volume at a distance from the micro-outlet is 36.6 mmHg, with the iso-line in A-D delineating the volume of tissue overlying the micro-outlet that experiences a difference up to ±2 mmHg during the $[O_2]$ challenges. Simulation results for high $[O_2]$ challenges at 12% $[O_2]$ in the chamber are shown at the middle of the outlet in the XZ plane (C) and at a depth of 10 µm into the tissue volume in the XY plane (D). The lower panel in A and C show a magnified view of the interface between the micro-outlet device and the overlying tissue.

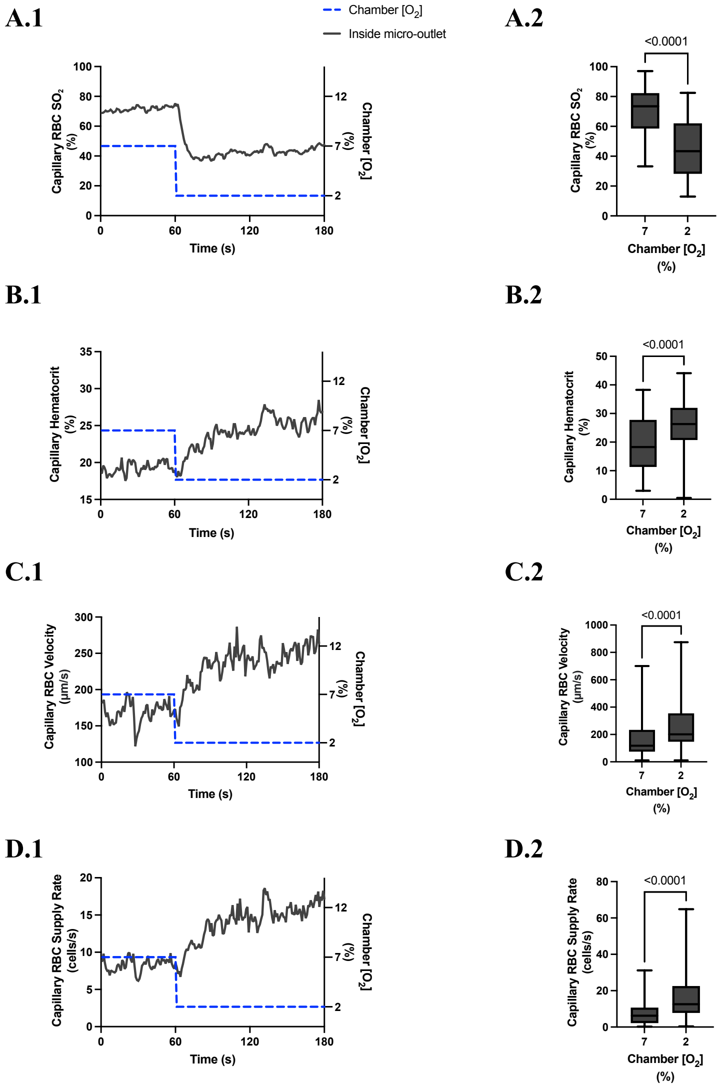

Figure S5

Capillary red blood cell (RBC) oxygen saturation (SO_2) and hemodynamic responses in capillaries directly overlying the 1000 µm micro-outlet edge in response to low oxygen concentration ([O_2]) challenges. [O_2] challenges began with one-minute baseline [O_2] at 7% followed by two minutes at 2%. Time series plots are displayed in panel A.1 – D.1 for mean capillary RBC SO_2 (A.1), hematocrit (B.1), velocity (C.1), and supply rate (D.1), for capillaries overlying the micro-outlet during low [O_2] challenges. For panel A.2 – D.2, the average was taken from the entire first minute at 7% and the last 15 seconds at 2% [O_2]. Panel A.2 – D.2 represent mean RBC SO_2 (n = 61 capillaries), hematocrit, velocity, and supply rate (n = 73 capillaries), respectively, for capillaries directly overlying the 200 µm micro-outlet. p values indicated in the figure with a $p < 0.05$ are considered significant. Box and whisker plots show minimum, median, maximum, and associated quartiles.

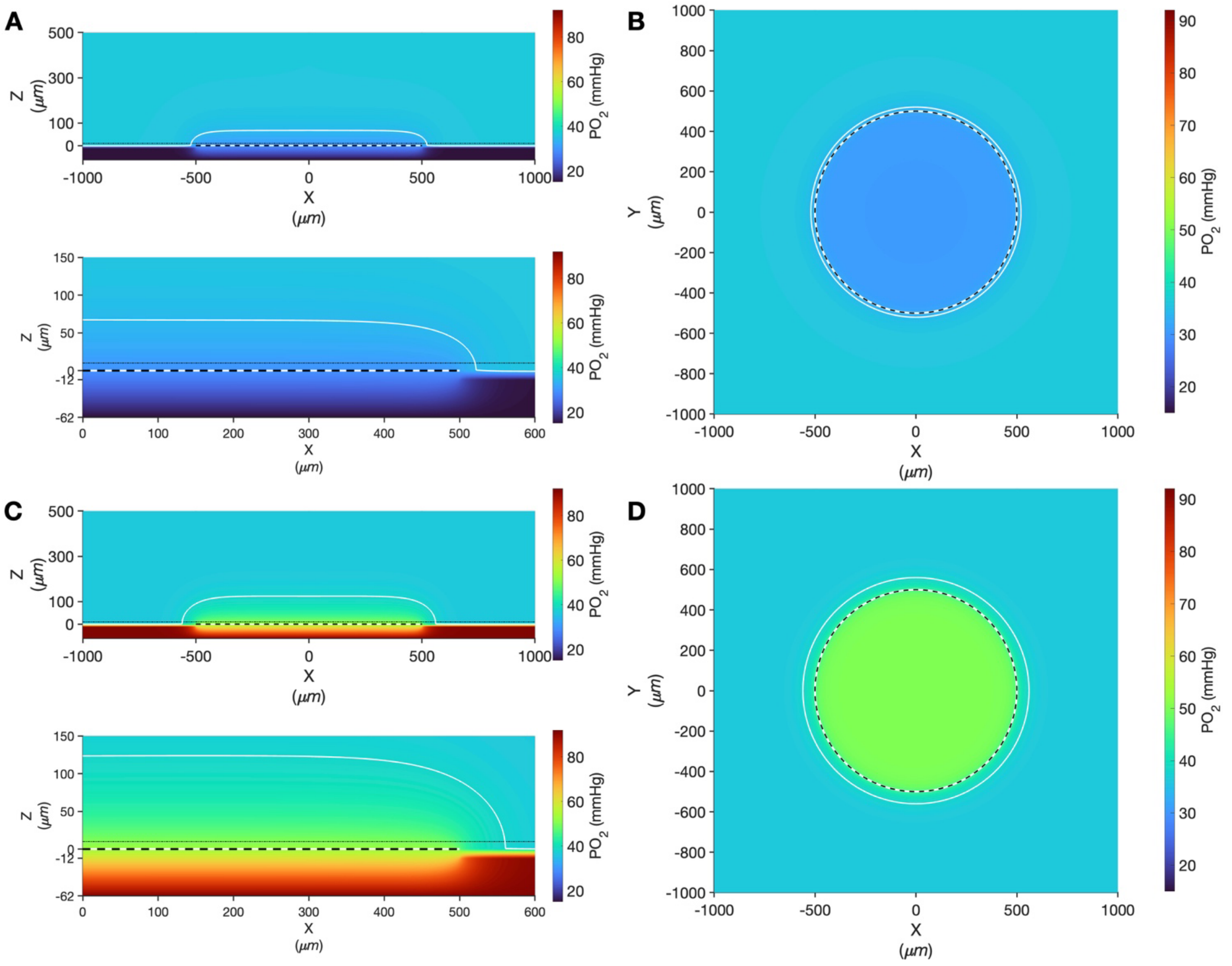

Figure S6

Oxygen transport simulation predicting tissue PO_2 resulting from diffusional exchange between the tissue and gas exchange chamber via a 1000 µm diameter thin-film micro-outlet device. In all panels the micro-outlet surface is indicated by the white and black dashed line. Simulation results for low oxygen concentration ($[O_2]$) challenges at 2% $[O_2]$ in the chamber are shown at the center of the outlet in the XZ plane (A). The dotted-dashed line in A indicates the location of the XY plane shown in panel B that is 10 µm into the tissue volume. Background tissue PO_2 within the volume at a distance from the micro-outlet is 36.6 mmHg, with the iso-line in A-D delineating the volume of tissue overlying the micro-outlet that experiences a difference up to ±2 mmHg during the $[O_2]$ challenges. Simulation results for high $[O_2]$ challenges at 12% $[O_2]$ in the chamber are shown at the middle of the outlet in the XZ plane (C) and at a depth of 10 µm into the tissue volume in the XY plane (D). The lower panel in A and C show a magnified view of the interface between the micro-outlet device and the overlying tissue.

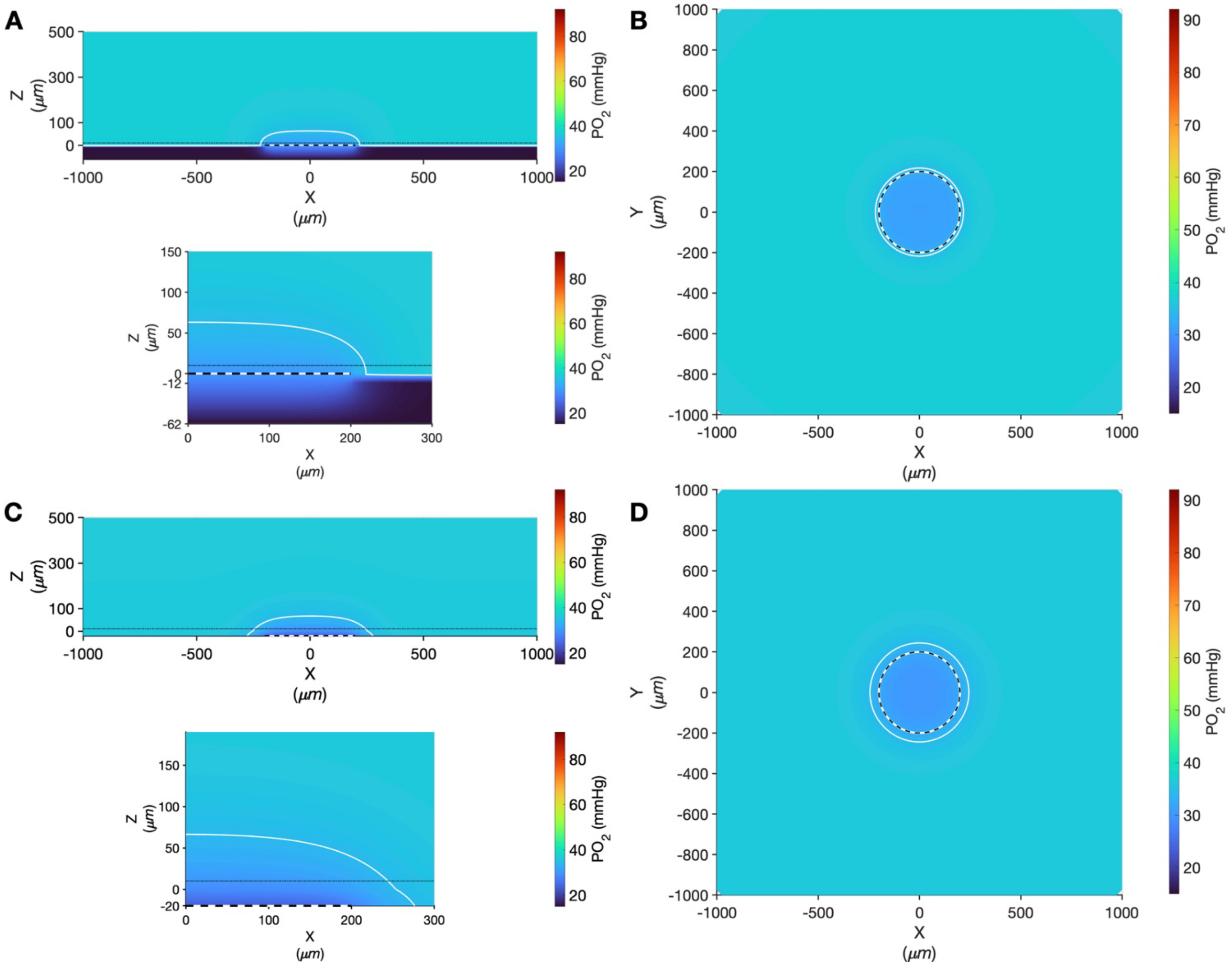

Figure S7

Comparison of low (7→ 2%) oxygen concentration ($[O_2]$) simulations for the current and previous micro-outlet geometries applying different orientations of the gas permeable layer with respect to the overlying tissue. The above simulations show the steady state result for a chamber $[O_2]$ of 2%. Our current device geometry (Panel A & B) places the gas permeable PDMS layer in contact with the gas exchange chamber flow channel, with the PVDC barrier layer in contact with the tissue, whereas our previous design (Panel C & D) using glass substrate placed the PDMS layer in contact with the tissue. Due to the high gas permeability of PDMS, the simulations of our previous device (Panel C) predict considerable radial spreading within the PDMS layer causing gas perturbations to alter tissue PO_2 at more than 3X the distance from the micro-outlet edge compared to our current design (53.0 vs 16.5 μm).

References

Asahi-Kasei Home Products Corporation (2024). PVDC wrapping film for food. Table showing comparison of performance by material. Available online at: https://www.asahi-kasei.co.jp/saran/global/asahiwrap/english/

Bentley, T. B., Meng, H., and Pittman, R. N. (1993). Temperature dependence of oxygen diffusion and consumption in mammalian striated muscle. Am J Physiol. 264 (6 Pt 2), H1825–H1830. doi:10.1152/ajpheart.1993.264.6.H1825

Goldman, D. (2008). A mathematical model of oxygen transport in intact muscle with imposed surface oscillations. Math. Biosci. 213, 18–28. doi:10.1016/j.mbs.2008.01.010

Honig, C., and Gayeski, T. (1982). Correlation of O_2 transport on the micro and macro scale. Int. J. Microcirc. Clin. Exp. 1, 367–380.

Mahler, M., Louy, C., Homsher, E., and Peskoff, A. (1985). Reappraisal of diffusion, solubility, and consumption of oxygen in frog skeletal muscle, with applications to muscle energy balance. J. Gen. Physiol. 86, 105–134. doi:10.1085/jgp.86.1.105

Merkel, T., Bondar, V., Nagai, K., Freeman, B., and Pinnau, I. (2000). Gas sorption, diffusion, and permeation in poly(dimethylsiloxane). J. Polym. Sci. Part B Polym. Phys. 38, 415–434. doi:10.1002/(sici)1099-0488(20000201)38:3<415::aid-polb8>3.0.co;2-z

Shiku, H., Saito, T., Wu, C.-C., Yasukawa, T., Yokoo, M., Abe, H., et al. (2006). Oxygen permeability of surface-modified poly(dimethylsiloxane) characterized by scanning electrochemical microscopy. Chem. Lett. 35, 234–235. doi:10.1246/cl.2006.234

Sullivan, S. M., and Pittman, R. N. (1984). In vitro O2 uptake and histochemical fiber type of resting hamster muscles. J. Appl. Physiol. 57, 246–253. doi:10.1152/jappl.1984.57.1.246
